# The Onset of ADL Difficulties and Changes in Health-Related Quality of Life

**DOI:** 10.1186/s12955-017-0792-8

**Published:** 2017-11-06

**Authors:** Wei Lyu, Fredric D. Wolinsky

**Affiliations:** 1 Department of Health Management and Policy, University of Iowa College of Public Health, 145 N. Riverside Dr., 100 College of Public Health Bldg., Room N269, Iowa City, Iowa, 52242-2007 USA; 2 Division of General Internal Medicine, Department of Internal Medicine, University of Iowa Carver College of Medicine, Iowa City, IA USA; 3 University of Iowa College of Nursing, Iowa City, IA USA

**Keywords:** Health Related Quality of Life, ADL, Medicare Advantage

## Abstract

**Background:**

The effect of the onset of difficulties with activities of daily living (ADLs) on the health-related quality of life (HRQoL) of older adults is not well understood. We identified strong longitudinal associations between ADL onset and HRQoL changes for older adults in Medicare Advantage Organizations (MAOs).

**Methods:**

We analyzed 473,282 age-eligible MAO beneficiaries in the 2008-2013 Medicare Health Outcomes Surveys (M-HOS) who reported no ADL difficulties at baseline and completed their two-year follow-ups in 2010-2015. The four HRQoL measures were the physical and mental health component scores (PCS and MCS) from the SF-12V, and the CDC’s counts of physically unhealthy and mentally unhealthy days (PUD and MUD) in the past month. Ordinary least squares (OLS) and zero-inflated negative binomial regressions were used.

**Results:**

The onset of difficulty/inability in bathing, dressing, eating, getting in/out of chairs, walking, and using the toilet significantly reduced PCS scores by 10.84, 11.29, 9.18, 8.98, 9.49 and 10.67 points, and MCS scores by 7.93, 8.72, 10.13, 5.34, 4.37 and 9.00 points, respectively. The onset of difficulty/inability in bathing, dressing, eating, getting in/out of chairs, walking, and using the toilet increased PUD days by 6.24, 6.83, 6.34, 4.93, 4.96 and 6.72 days, and MUD days by 3.00, 3.19, 3.54, 2.26, 2.07 and 3.27 days, respectively.

**Conclusions:**

There is robust evidence that the onset of ADL difficulties/inabilities significantly and substantially reduced age-eligible MAO beneficiaries’ HRQoL. Prevention strategies focused on ADLs would benefit the performance of MAOs.

## Background

About 13% of Americans were 65 years old or older in 2010, but this percentage will increase to more than 20% by 2030 [1]. Because older adults are more likely to have reduced functional ability and greater difficulties performing daily activities of living (ADLs), their health-related quality of life (HRQoL) has become a major public health concern. HRQoL is a multidimensional concept that measures how individuals perceive their physical and mental well-being [2]. The Center for Disease Control and Prevention (CDC) has, in fact, targeted twelve objectives (OA-1 to OA-12) in *Healthy People 2020* to improve the HRQoL of older adults. One specific objective (OA-5) is to “reduce the proportion of older adults who have moderate to severe functional limitations” [3]. About one-third (32.2%) of older Americans either had difficulty with or were unable to perform at least one ADL or lived in long-term care facilities in 2011. *Healthy People 2020* sets a health policy goal of reducing that number to 26.4% by 2020.

To develop appropriate programs and interventions for reaching this *Healthy People 2020* goal, a better understanding of the relationships between functional limitations and HRQoL is needed. Previous studies have shown that functional limitations are either directly or indirectly associated with poorer HRQoL. For example, using baseline data from the 2004 Medicare Health Outcome Survey (M-HOS) it has been shown that for every additional difficulty with or inability to perform an ADL, the number of physically unhealthy days (PUD) in the last month increased by 2.1 days and the number of mentally unhealthy days (MUD) increased by 0.9 days, even after adjusting for basic demographic factors and chronic conditions [4]. A similar study used the same 2004 M-HOS baseline data but with follow-up data two years later, and the researchers found longitudinal associations that about 10.69 additional PUDs and 3.42 additional MUDs are associated with 3 additional reported ADL limitations after two years [5]. Another study showed that the decline of global cognitive function scores derived from four measures of memory and perceptual speed before the onset of a new difficulty with or inability to perform ADL were 0.05 units per year, but in the next year they increased to 0.08 units per year, in comparison to the baseline mean cognitive score of 0.36 points [6]. Other studies have looked at how ADL limitations affect HRQoL, but only among those with certain chronic conditions like dementia, cognitive impairment, Parkinson’s disease, or survivors of intensive care units after a stroke [7–9]. It has also been shown that people with functional limitations benefit less from their socioeconomic status which might otherwise have increased their HRQoL [10].

While these studies provide evidence that ADLs are associated with HRQoL among older adults, for the most part they involved restricted samples that limit their generalizability, and used only one or two years of cross-sectional data. Our study will fill in these gaps in the evidentiary base by investigating the effects of the onset of difficulty with or the inability to perform ADLs on HRQoL in a nationally representative sample of older adults who were enrolled in Medicare Advantage Organizations (MAOs; Medicare Part C). We use seven sequential cohorts from the M-HOS each of which includes baseline and two-year follow-up surveys. This allows us to move beyond the association between ADLs and HRQoL in cross-section, toward the estimation of longitudinal associations. To do so we first selected an analytic sample of MAO beneficiaries who reported no difficulties with or inabilities to perform ADLs during their baseline surveys. Then, we tested the effects of the onset of difficulties with or inabilities to perform ADLs that the MAO beneficiaries reported at their two-year follow-up surveys on changes in their HRQoL over that same two-year period. We do this using four well-established, reliable, and valid HRQoL measures.

## Methods

### Data sources and study population

We used data from the M-HOS Limited Data Set (LDS) for all of our analyses. The M-HOS was developed by the Centers for Medicare and Medicaid Services (CMS) to monitor and evaluate the quality of care provided to beneficiaries enrolled in MAOs [11]. Among MAOs with at least 500 covered lives, a random sample is selected every year consisting of 500-1200 beneficiaries based on the size of the MAO’s enrollment. Sampled beneficiaries in a given year receive a baseline survey and a follow-up survey two years later. Thus, the M-HOS is a cross-sequential series of cohorts empaneled with baseline and two-year follow-up surveys. Surveys are usually mailed to the sample beneficiaries with telephone follow-ups when necessary to harvest missing data for partially completed and returned surveys, or for global nonresponse.

**Fig. 1:**
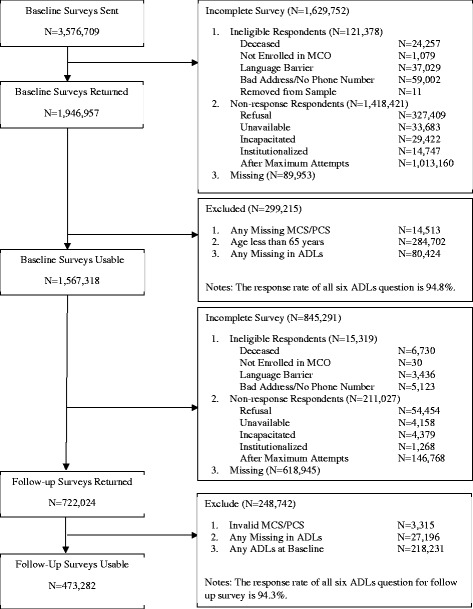
Sample Digram

As shown in Fig. 1, our analytic sample consists of the 473,282 MAO beneficiaries who were 65 years old or older at their baseline surveys, reported having no difficulties with or inabilities to perform ADLs at baseline, completed their two-year follow up surveys, and had no missing data on the HRQoL outcomes, the focal variable (ADL onset) or the covariates. We used data from seven consecutive M-HOS annual cohorts. Their baseline data was obtained from 2007 to 2013 and their follow-up data was obtained from 2009 to 2015.

### HRQoL Outcomes

There are four HRQoL outcomes. The first two are the physical and mental health component scores (PCS and MCS) from the Medical Outcomes Study Short-Form 12-item questionnaire for veterans (SF-12V). The PCS and MCS range from 0 to 100 and are nationally normed to have a mean of 50 and a standard deviation of 10. The second two HRQoL measures are the CDC’s measures of the number of physically (“Now thinking about your physical health, which includes physical illness and injury, how many days during the past 30 days was your physical health not good?”) or mentally (“Now thinking about your mental health, which includes stress, depression, and problems with emotions, how many days during the past 30 days was your mental health not good?”) unhealthy days (PUD and MUD) during the last 30 days [12]. The PUD and MUD counts range from 0 to 30.

In assessing subjective outcomes like the HRQoL, the validity and reliability of these measures are crucial. PCS and MCS scores, which were originally derived from the SF-36, are the two most widely used HRQoL measures for the general population, and have well-established reliability, validity, and sensitivity to change [13]. Even though the PCS and MCS in our study are derived from SF-12V, previous studies have shown high correlations with, and strong agreement between SF-36 MCS/PCS and SF-12 MCS/PCS in the general population. One study examined the actual scores across 17 populations and found that the average SF-36 and SF-12 PCS and MCS scores differed by less than one point, which suggests that the MCS/PCS from the SF-12 are as valid as those from SF-36 [14]. The CDC healthy days measures provide simple but comprehensive HRQoL summary measures which are reliable, valid, and sensitive to change in measuring physical and mental well-being over time [12]. Previous studies report high levels of agreement between these measures and the assessment of health professionals [12, 15, 16].

### Focal variables

The focal independent variables in our study are the onset of ADL difficulties or the inability to perform ADLs. The six ADLs are bathing, dressing, eating, getting in or out of chairs, walking, and using the toilet. Respondents were asked to answer “Because of a health or physical problem, do you have any difficulty doing the following activities without special equipment or help from another person?” We first recoded each ADL item into a binary indicator coded 1 for “Yes, I have difficulty” or “I am unable to do this activity” responses, and 0 for “No, I do not have difficulty”. Thus, these binary indicators reflect the onset of these ADL limitations between baseline (when none of the respondents reported having any ADL limitations) and the two-year follow-up. The response rate for answering all six ADL questions at baseline was 94.8% and at the two-year follow-up was 94.3%.

We model the effects of the onset of difficulty with or the inability to perform ADLs on the SF-12V PCS and MCS scores using two different approaches. First, for each ADL we analyzed how the onset of difficulty/inability between the baseline and follow-up surveys affected the SF-12V MCS or PCS scores at follow-up. The onset of difficulty/inability with each ADL is then regressed on these HRQoL outcomes, adjusting for the covariates. This yields six models (one for each ADL difficulty/inability onset) for each of the two outcomes (PCS and MCS). In the second approach, we analyze the effect of the count of all six ADL difficulty/inability onset indicators on the SF-12V MCS or PCS scores at follow-up. We model the effects of the onset of difficulty with or the inability to perform ADLs on the CDC’s PUD or MUD HRQoL measures using the latter approach. That is, the count of all six ADL difficulty/inability onset indicators is regressed on the PUD or MUD days, adjusting for the covariates.

### Covariates

Based on prior studies of the effects of the association between ADL difficulty/inability and HRQoL, we adjust for a variety of baseline covariates that might serve as potential confounders [4, 8, 17, 18]. As shown in Table 1, these covariates included gender, race/ethnicity, education, annual income, marital status, medical diagnoses, housing status, body mass index (BMI), smoking status, dual-energy X-ray absorptiometry (DXA) testing, urinary incontinence, and baseline health. We also included a time-dependent secular trend measure which is set to 1 for 2007 through 7 for 2013.

**Table 1:** Descriptive Statistics

	Mean/Rates	SD
Health-related Quality of Life Outcomes (Dependent Variables)		
PCS	43.79	10.13
MCS	54.75	8.91
PUD	3.55	8.50
MUD	2.17	6.92
Focal Variables		
ADL Items		
Bathing	0.04	0.20
Dressing	0.03	0.18
Eating	0.02	0.13
Getting In/Out of Chairs	0.09	0.28
Walking	0.14	0.35
Using the Toilet	0.02	0.15
ADL Counts		
0	0.82	0.38
1	0.09	0.29
2	0.05	0.21
3	0.02	0.12
4	0.01	0.09
5	0.01	0.08
6	0.01	0.09
Covariates		
Baseline HRQoL Measures		
PCS	45.79	8.91
MCS	55.38	8.20
PUD	2.37	7.11
MUD	1.71	6.39
Male	0.57	0.50
Race Category		
White	0.87	0.34
Black	0.07	0.26
Other	0.02	0.15
Asian	0.02	0.15
Hispanic	0.02	0.12
American Native	0.00	0.04
Unknown	0.00	0.05
Education Category		
<High School	0.19	0.39
High School or GED	0.35	0.48
Some College or 2 year degree	0.23	0.42
Four Year College Degree or More	0.21	0.41
Missing	0.02	0.13
Income Category		
<$10,000	0.08	0.27
$10,000-$19,999	0.17	0.38
20000-29999	0.17	0.37
30000-49999	0.21	0.41
>50000	0.19	0.39
Don't Know	0.09	0.28
Missing	0.10	0.29
Marital Status		
Married	0.61	0.49
Divorced	0.12	0.32
Separated	0.01	0.10
Widowed	0.22	0.42
Never Married	0.03	0.18
Missing	0.02	0.12
Medical History		
Hypertension	0.61	0.49
Angina/Coronary Artery Disease	0.10	0.30
Congestive Heart Failure	0.04	0.20
Myocardial Infarction Question	0.07	0.26
Other Heart Conditions Question	0.17	0.38
Stroke	0.05	0.21
COPD	0.11	0.31
Inflammatory Bowel Disease	0.04	0.18
Arthritis of Hip/Knee	0.30	0.46
Arthritis of Hand/Wrist	0.29	0.45
Osteoporosis	0.17	0.38
Sciatica	0.16	0.36
Diabetes	0.20	0.40
Any Cancer	0.14	0.35
Housing Category		
Owned or being bought by you	0.76	0.43
Owned or bought by someone in your family	0.05	0.22
Rented for money	0.11	0.32
Live without payment of rent	0.01	0.12
None of the above	0.04	0.18
Missing	0.03	0.16
BMI Category	0.76	0.43
Underweight ( < 20)	0.05	0.21
Normal (20-24)	0.31	0.46
Overweight (25-29)	0.41	0.49
Obese (30-34)	0.16	0.37
Morbid Obesity (>=35)	0.06	0.23
Missing	0.02	0.15
Current Smoker	0.08	0.28
Bone Density Test for Osteoporosis	0.49	0.50
Urine Leakage	0.30	0.46
Talked with Doctor About Physical Activities	0.51	0.50
Benefit Durantion Less than 1 Year	0.30	0.46
Plan Duration Less than 5 Year	0.36	0.48
Year Trend	4.05	1.93

### Analysis

The general model for our analyses is:

$$ {\mathit{\mathsf{Outcome}}}_{\mathit{\mathsf{it}}}=\mathit{\mathsf{\alpha}}+{\mathit{\mathsf{\beta}}}_{\mathsf{1}}\mathit{\mathsf{AD}}{\mathit{\mathsf{L}}}_{\mathit{\mathsf{it}}}+{\boldsymbol{\mathsf{X}}}_{\boldsymbol{\mathsf{it}}}{\boldsymbol{\mathsf{\boldsymbol{\beta}}}}_{\mathsf{2}}+{\mathit{\mathsf{\beta}}}_{\mathsf{3}}\mathit{\mathsf{Tren}}{\mathit{\mathsf{d}}}_{\mathit{\mathsf{t}}}+{\mathit{\mathsf{e}}}_{\mathit{\mathsf{it}}} $$

where *Outcome*_*it*_ is one of the HRQoL measures (PCS, MCS, PUD, or MUD) of individual i at time t, *ADL*_*it*_ is the focal ADL difficulty/inability onset measure, **X**_**it**_ is a vector of baseline characteristics, and Trend_t_ is the secular trend indicator. For the SF-12V MCS and PCS scores, we apply ordinary least square (OLS) regression, which offers the mean effect of ADL onset on individuals’ physical and mental health. Because 67.9% and 77.6% of the individuals in our sample reported that they had zero physically unhealthy or mentally unhealthy days in the last 30 days, we use zero-inflated negative binominal (ZINB) regressions to estimate the effects of the focal ADL difficulty/inability onset measures on the number of CDC PUD and MUD days. ZINB models the excess number of zeros in addition to allowing the count data to be skewed and over-dispersed. It is a two-part process that combines a logistic regression model that predicts the probability of having non-zero unhealthy days with a negative binomial regression model that predicts the frequency of the unhealthy days [19]. We used a ZIP likelihood-ratio test to compare ZINB with the alternative approach of using zero-inflated Poisson regression models [20] and a Vuong test to compare ZINB with the alternative approach of using regular negative binomial regression. Both tests were statistically significant indicating that the ZINB was the preferred approach [21]. All standard errors were clustered within MAOs to account for potential within-cluster correlation.

## Results

Table 1 provides means and standard deviations of the dependent and independent variables. For the analytic sample, all of whom had reported having no ADL difficulties/inabilities at baseline, the average baseline SF-12V PCS and MCS scores were 45.79 and 55.38, respectively. At the two-year follow-up the average PCS and MCS scores had significantly (p<0.05) decreased to 43.79 and 54.75, respectively. For the CDC unhealthy days measures, at baseline the mean number of PUD and MUD days were 2.37 days and 1.71 days, respectively. At the two-year follow-up the mean number of PUD and MUD days had significantly (p<0.05) increased to 3.55 days and 2.17 days, respectively. Limitations in ADLs are likely not the only source for these HRQoL declines, which could include the two additional years of the aging process. Between their baseline and two-year follow-up surveys, 17.8% of the individuals in our analytic sample developed at least one of the six ADLs. In descending prevalence, the onset of difficulty/inability in walking was 14.3%, of getting in/out chairs was 8.5%, of bathing was 4.4%, of dressing was 3.3%, of using the toilet was 2.4%, and of eating was 1.6%. Among those who suffered the onset of one or more ADL difficulties/inabilities, the mean number of such ADL difficulties/inabilities was 1.9.

Panels A and B of Table 2 show the OLS results for the two approaches to estimating the effects of the ADL onset indicators on the SF-12V PCS and MCS scores, and the full results are listed in the online Appendix. All of these models adjust for the covariates discussed above, and the coefficients for those covariates are shown in the online Appendix. As shown in Panels A and B of Table 2, the effects of the onset of difficulty/inability with each ADL are significant and negative, indicating declining HRQoL. Specifically, the onset of difficulty/inability in bathing, dressing, eating, getting in/out of chairs, walking, and using the toilet reduced the PCS scores by 10.84, 11.29, 9.18, 8.98, 9.49 and 10.67 points (panel A), and the MCS scores by 7.93, 8.72, 10.13, 5.34, 4.37 and 9.00 points (Panel B). When the count of ADL difficulty/inability onset is regressed on the PCS scores at follow-up (Panel A), compared to respondents having no ADL difficulty/inability onset, those who developed one or more ADL difficulties/inabilities had PCS scores reductions that ranged from 7.10 points to 16.53 points as the ADL onset counts increased from one to five and the effect reduced to 13.36 for six ADL onset counts. When the count of ADL difficulty/inability onset is regressed on the MCS scores at follow-up (Panel B), compared to respondents having no ADL difficulty/inability onset, individuals who developed one or more ADL difficulties/inabilities had reductions in MCS scores that ranged from 2.29 points for one ADL to 13.36 points for all six ADL items. Note that when comparing the effects of the count of ADL difficulties/inabilities on the PCS vs. MCS scores, the MCS effects were smaller, but by the time that the ADL onset count reached six, the reductions in PCS and MCS were almost the same. This indicates that the onset of ADL difficulties/inabilities are smaller but more independent for the MCS vs. the PCS, and therefore cumulate faster.

**Table 2:** OLS estimates of ADL measures on PCS and MCS

Panel A Physical Component Score (PCS)								
ADL Item							ADL Count	
Bathing	-10.84***						1	-7.10***
	(0.09)							(0.05)
Dressing		-11.29***					2	-9.62***
		(0.10)						(0.06)
Eating			-9.18***				3	-12.26***
			(0.14)					(0.11)
Getting In/Out of Chairs				-8.98***			4	-14.56***
				(0.06)				(0.16)
Walking					-9.49***		5	-16.53***
					(0.05)			(0.18)
Using the Toilet						-10.67***	6	-13.36***
						(0.11)		(0.20)
Panel B Mental Component Score								
Bathing	-7.93***						1	-2.29***
	(0.09)							(0.05)
Dressing		-8.72***					2	-4.28***
		(0.10)						(0.07)
Eating			-10.13***				3	-6.71***
			(0.15)					(0.13)
Getting In/Out of Chairs				-5.34***			4	-9.40***
				(0.06)				(0.17)
Walking					-4.37***		5	-11.80***
					(0.05)			(0.22)
Using the Toilet						-9.00***	6	-13.36***
						(0.11)		(0.22)

Panels A and B of Table 3 shows the ZINB results of the onset of difficulty/inability with each of the ADL onset measures on the two CDC unhealthy days outcomes, and the full results are shown in the online Appendix. These are the average marginal effects derived from the Incidence Rate Ratios (IRRs) and have a more practical interpretation. Panel A shows the average marginal effects of ADL difficulty/inability onset on PUD days and Panel B shows the effects on MUD days. The onset of difficulty/inability in bathing, dressing, eating, getting in/out of chairs, walking, and using the toilet increased PUD days by 6.24, 6.83, 6.34, 4.93, 4.96 and 6.72 days, and MUD days by 3.00, 3.19, 3.54, 2.26, 2.07 and 3.27 days, respectively. As with the PCS and MCS, this indicates that the onset of difficulty/inability on any one ADL onset has smaller effects on MUD days than on PUD days. Individuals who developed one or more ADL difficulties/inabilities had increased PUD days that ranged from 4.36 to 16.35 days as the ADL count rose from one through five compared to those with no ADL difficulty/inability onset. This effect reduced to 14.22 when individuals developed all six ADL limitations. As the ADL count rose from 1 through 6, the number of MUD days increased from 1.39 to 9.80 days compared to those with no ADL difficulty/inability onset.

**Table 3:** Marginal Effects of On-set ADLs on Unhealthy Days by using Zero-inflated Negative Binomial Regression

Panel A Physically Unhealthy Days (PUD)								
ADL Item							ADL Count	
Bathing	6.24***						1	4.36***
	(0.06)							(0.06)
Dressing		6.83***					2	7.26***
		(0.09)						(0.08)
Eating			6.34***				3	10.59***
			(0.12)					(0.16)
Getting In/Out of Chairs				4.93***			4	13.88***
				(0.04)				(0.23)
Walking					4.96***		5	16.35***
					(0.04)			(0.25)
Using the Toilet						6.72***	6	14.22***
						(0.10)		(0.25)
Panel B Mentally Unhealthy Days (MUD)								
Bathing	3.00***						1	1.39***
	(0.03)							(0.04)
Dressing		3.19***					2	2.56***
		(0.04)						(0.06)
Eating			3.54***				3	4.35***
			(0.05)					(0.12)
Getting In/Out of Chairs				2.26***			4	6.36***
				(0.03)				(0.17)
Walking					2.07***		5	8.54***
					(0.02)			(0.24)
Using the Toilet						3.27***	6	9.80***
						(0.04)		(0.22)

Notes: Panel A adjusted for covariates listed in Table 1 except MCS, PUD and MUD. Panel B adjusted for covariates listed in Table 1 except PCS, PUD and MUD. *p<0.1, **p<0.05, ***p<0.01

In addition to ADL limitations onset, people with better socioeconomic status, like higher income and education, generally had higher scores on all four HRQoL measures. The associations between race/ethnicity and HRQoL are different between physical and mental HRQoL measures. For example, in comparison to whites, blacks have higher scores on physical measures (PCS and PUD) but lower scores on mental measures (MCS and MUD). And for other minorities, we find negative associations across all four HRQoL measures. Among the fourteen medical conditions we adjusted for in our analyses, chronic obstructive pulmonary disease (COPD), arthritis of the hip/knee and congestive heart failure are the three conditions that worsen older adults’ physical HRQoL the most. For mental HRQoL, the three conditions that worsen scores the most are inflammatory bowel disease, congestive heart failure, and stroke. Risk behaviors, like smoking and obesity, are negatively associated with all four HRQoL measures.

## Discussion

Using a cross-sequential design involving seven annual cohorts yielding 473,282 MAO beneficiaries who reported no difficulties with or inabilities to perform ADLs at the time of their baseline survey, we evaluated the effects of the onset of ADL difficulties/inabilities on their HRQoL by their two-year follow-up survey, after adjusting for baseline covariates. To our knowledge, this is the first study to address this question longitudinally using a large sample derived from seven consecutive randomly selected cohorts. HRQoL was measured by the SF-12V PCS and MCS scores which were analyzed using OLS regression models, and the CDC MUD and PUD unhealthy days measures which were analyzed using ZINB regression models with the results expressed as overall marginal effects.

Our results indicate consistent and strong longitudinal associations such that the onset of ADL difficulties/inabilities decreased HRQoL for Medicare beneficiaries who were in the MAOs. In terms of the count of ADL limitations, our results are similar to Barile’s analysis of the 2004-2006 M-HOS which reported that three additional ADL limitations were associated with 10.69 PUD days and 3.42 MUD days [5], vs. our findings of 10.59 PUD days and 4.35 MUD days . Another interesting finding is that when individuals developed all six ADLs, the reduction in the physical HRQoL measures, the PCS and PUD, is somewhat smaller than it was for individuals who developed five ADLs. Among people who developed five ADLs, about 77% had no limitation in eating. This is consistent with the original hierarchy of ADL items identified by Katz and his colleagues[22]. That is, eating is one of the last ADL items “to go”. Thus, people who develop eating limitations are much more likely to be institutionalized. Indeed, in these data those who developed all six ADLs were twice as likely to be institutionalized, which, of course, increased their access to formal care providers to help them cope, which likely led to their better HRQoL scores.

A potential concern of our study, especially for the PCS and MCS analyses, is that the items in the SF-12V measure both physical and mental functions, which might make PCS and MCS less accurate measures of HRQoL [23, 24]. However, we believe this was not a major issue in our study. We followed the path taken in the previous literature and did an item-by-item analysis of the SF-12 for each individual ADL onset [23, 24]. The logic of this analysis was that by regressing each ADL onset on each SF-12 item, we separately explained how each ADL onset affects each SF-12 item. The SF-12 items were recoded to have same direction that higher scores represent worse HRQoL. The results listed in Appendix Table 5 show the standardized (beta) coefficients, which indicate that each ADL onset is positively associated with each SF-12 item. This is consistent with our findings that each ADL onset worsens both PCS and MCS scores. On the one hand, this is not surprising because ADL onset affects people’s physical health, especially the physical functioning domain. However, our item-by-item analysis shows that relative to the physical functioning domain, ADL onset had a larger effect on the role-physical, bodily pain and social domains. This implies that the effect of ADL onset on HRQoL is not driven by physical functioning, even though ADL onset seems to affect physical functioning more. On the other hand, we also utilized the CDC unhealthy days items which are better instruments for measuring HRQoL because they are independent of the individuals’ functional limitations [25]. And the estimations on the CDC unhealthy days were quite similar to those for the SF-12V PCS and MCS scores.

To better understand the difference across the SF-12 items and CDC unhealthy days items in our sample, we conducted two exploratory factor analyses using the principal component methods with oblique rotation. The first factor analysis had 14 variables including all SF-12 items and the two CDC unhealthy days items (PUD and MUD), and the results listed in the Panel A of Appendix Table 6. The overall Kaiser-Meyer-Olkin (KMO) value is 0.92 which indicates good sampling adequacy, and the correlation between two factors is a robust 0.54. Our second factor analysis had four variables including the PCS, MCS, PUD, and MUD, and the results are listed in Panel B of Appendix Table 6. The overall KMO value here is 0.64 which still indicates the sample is adequate, and the correlation between two factors is lower at -0.34.

For both factor analyses, a simple factor structure was observed such that these variables are loaded onto two underlying factors—a physical health factor and a mental health factor. We found that the MUD had similar factor loadings with the SF-12 items which measure mental health and the MCS, while the PUD had somewhat different factor loadings from the SF-12 items which measure physical health and the PCS. Therefore, MUD measures a latent mental health factor similar to the SF-12 mental health items and the MCS, but PUD measures a somewhat different latent physical health factor than the relevant SF-12 physical health items and the PCS. The main reason for the differences between PUD and the SF-12 items in measuring physical health might be that the PUD does not include questions about individual’s physical functioning while the SF-12 does [25].

Our results highlight preventing ADL difficulty/inability onset as a specific intervention target for improving older adults’ HRQoL at the MAO level. The reason is that in 2008, CMS implemented the 5-star rating system to provide beneficiaries information about the quality of MAOs and to assist beneficiaries in the plan selection process. In 2011, payment bonuses were awarded to MAOs that received 4 or more stars [26]. The SF-12V PCS and MCS scores are two of the five M-HOS measures that are used to calculate the 5-star ratings for each MAO. Therefore, MAOs would likely benefit by preventing ADL difficulty/inability onset in the first place (primary prevention), or by screening for the onset of ADL difficulty/inability onset for subsequent remediation (secondary prevention). Interventions to improve the management of some chronic conditions, like COPD and heart disease, would be another approach to increase HRQoL among older adults in MAOs.

In closing, we note that this study has several limitations. First, only MAO beneficiaries were included, who may be more likely to have better socioeconomic status and be healthier than beneficiaries enrolled in traditional Medicare (Parts A and B), creating the potential for selection bias. Furthermore, by necessity MAO beneficiaries who did not complete their follow-up interview for any reason were also excluded, creating the potential for attrition bias. Thus, these results cannot be generalized to other populations like older adults in traditional Medicare, or to younger adults, or those lost to follow-up. Therefore, further research is needed that includes fee-for-service Medicare beneficiaries, the non-elderly population, and achieves lower attrition. Second, even though the evidence is clear and compelling that the onset of difficulties/inabilities in performing ADLs substantially reduced HRQoL among these MAO beneficiaries, those HRQoL reductions only adjusted for the baseline covariates and HRQoL measures, rather than changes in those baseline covariates. Furthermore, we cannot identify whether these limitations are acute with temporary effects, or chronic with lifelong effects. With only two time points (baseline and the two-year follow-up), however, it would be difficult to interpret dynamic adjustments (changes in covariates over the two-year period). Moreover, there might be unobserved changes in health conditions that also contribute to both the onset of ADL limitations and the declines in HRQoL. A mixed methods approach using semi-structured interviews with representative samples of those who faced ADL onset and whose HRQoL either declined, remained the same, or improved might further our understanding of the underlying processes involved here.

## Conclusion

The onset of ADL difficulties/inabilities among these MAO beneficiaries reduced HRQoL on both the physical and mental dimensions for both the SF-12V PCS and MCS scores and the CDC’s PUD and MUD days measures after adjusting for the baseline covariates. The evidence for this was robust. The onset of difficulty/inability for each of the six ADLs was associated with an 8.98 to 11.29 point decline in the SF-12V PCS scores and a 5.34 to 9.00 point decline in the MCS scores, as well as an increase of 4.93 to 6.83 PUD days and 2.07 to 3.54 MUD days. Compared to MAO beneficiaries with no ADL onset, going from the onset of one ADL difficulty/inability to six led to declines ranging from 7.10 to 13.36 points for the SF-12V PCS scores and 2.29 to 13.36 points for the MCS scores, and from 4.36 to 14.22 days for the CDC PUD days and from 1.39 to 9.80 days for the CDC MUD days.

## Appendix 1

**Table 4:** Full OLS estimates of ADL measures on PCS

	Each ADL						ADL Counts
	(1)	(2)	(3)	(4)	(5)	(6)	(7)
	PCS	PCS	PCS	PCS	PCS	PCS	PCS
N	473282	473282	473282	473282	473282	473282	473282
R-Squared	0.435	0.428	0.401	0.447	0.486	0.414	0.507
PCS_Baseline	0.555***	0.564***	0.581***	0.543***	0.502***	0.574***	0.488***
	(0.00)	(0.00)	(0.00)	(0.00)	(0.00)	(0.00)	(0.00)
Male	-0.0951**	0.00173	-0.0843*	-0.0110	0.00990	-0.0482	0.0795**
	(0.03)	(0.03)	(0.04)	(0.03)	(0.03)	(0.03)	(0.03)
Race Category							
White	0	0	0	0	0	0	0
	(.)	(.)	(.)	(.)	(.)	(.)	(.)
Black	0.278***	0.253***	0.217***	0.0699	-0.0657	0.218***	0.0262
	(0.05)	(0.05)	(0.05)	(0.05)	(0.05)	(0.05)	(0.05)
Other	0.188*	0.214*	0.259**	0.0882	0.116	0.254**	0.0287
	(0.09)	(0.09)	(0.09)	(0.09)	(0.09)	(0.09)	(0.09)
Asian	-0.331***	-0.323***	-0.293**	-0.430***	-0.409***	-0.234**	-0.433***
	(0.08)	(0.09)	(0.10)	(0.09)	(0.09)	(0.09)	(0.09)
Hispanic	-0.220*	-0.140	-0.246*	-0.156	-0.235*	-0.219*	-0.0784
	(0.10)	(0.10)	(0.10)	(0.10)	(0.09)	(0.10)	(0.09)
American Native	-0.895*	-0.891**	-0.952**	-0.899**	-0.803**	-0.990**	-0.756*
	(0.36)	(0.34)	(0.36)	(0.35)	(0.30)	(0.36)	(0.30)
Unknown	0.863***	0.889***	0.839***	0.710**	0.570**	0.797***	0.601**
	(0.21)	(0.22)	(0.22)	(0.22)	(0.21)	(0.22)	(0.20)
Education Category							
<High School	0	0	0	0	0	0	0
	(.)	(.)	(.)	(.)	(.)	(.)	(.)
High School or GED	0.454***	0.481***	0.540***	0.531***	0.468***	0.519***	0.403***
	(0.04)	(0.04)	(0.04)	(0.04)	(0.04)	(0.04)	(0.04)
Some College or 2 year degree	0.698***	0.746***	0.784***	0.809***	0.740***	0.781***	0.697***
	(0.04)	(0.04)	(0.04)	(0.04)	(0.04)	(0.04)	(0.04)
Four Year College Degree or More	1.033***	1.083***	1.116***	1.158***	1.049***	1.116***	1.032***
	(0.05)	(0.05)	(0.05)	(0.05)	(0.05)	(0.05)	(0.05)
Missing	0.129	0.172	0.219	0.229	0.135	0.216	0.118
	(0.13)	(0.13)	(0.13)	(0.13)	(0.12)	(0.13)	(0.12)
Income Category							
<$10,000	0	0	0	0	0	0	0
	(.)	(.)	(.)	(.)	(.)	(.)	(.)
$10,000-$19,999	0.0469	0.0951	0.135*	0.114*	0.116*	0.104	0.00904
	(0.05)	(0.05)	(0.05)	(0.05)	(0.05)	(0.05)	(0.05)
20000-29999	0.320***	0.377***	0.437***	0.390***	0.370***	0.406***	0.238***
	(0.05)	(0.06)	(0.06)	(0.06)	(0.05)	(0.06)	(0.05)
30000-49999	0.662***	0.736***	0.799***	0.726***	0.687***	0.765***	0.536***
	(0.05)	(0.05)	(0.05)	(0.05)	(0.05)	(0.05)	(0.05)
>50000	1.177***	1.243***	1.320***	1.204***	1.158***	1.278***	0.993***
	(0.06)	(0.06)	(0.06)	(0.06)	(0.06)	(0.06)	(0.05)
Don't Know	0.434***	0.475***	0.493***	0.417***	0.404***	0.492***	0.316***
	(0.06)	(0.06)	(0.06)	(0.06)	(0.06)	(0.06)	(0.06)
Missing	0.618***	0.680***	0.746***	0.668***	0.638***	0.727***	0.476***
	(0.06)	(0.06)	(0.06)	(0.06)	(0.06)	(0.06)	(0.05)
Marital Status							
Married	0	0	0	0	0	0	0
	(.)	(.)	(.)	(.)	(.)	(.)	(.)
Divorced	0.413***	0.384***	0.456***	0.416***	0.425***	0.434***	0.390***
	(0.04)	(0.04)	(0.04)	(0.04)	(0.04)	(0.04)	(0.04)
Separated	0.335*	0.367**	0.429**	0.408**	0.434**	0.408**	0.408**
	(0.14)	(0.14)	(0.14)	(0.14)	(0.14)	(0.14)	(0.13)
Widowed	-0.255***	-0.348***	-0.344***	-0.260***	-0.105***	-0.326***	-0.105***
	(0.03)	(0.03)	(0.03)	(0.03)	(0.03)	(0.03)	(0.03)
Never Married	0.650***	0.574***	0.638***	0.641***	0.641***	0.630***	0.647***
	(0.06)	(0.06)	(0.06)	(0.06)	(0.06)	(0.06)	(0.05)
Missing	0.108	0.0186	0.101	0.194	0.247	0.0769	0.252
	(0.14)	(0.14)	(0.14)	(0.14)	(0.14)	(0.14)	(0.13)
Medical History							
Hypertension	-0.622***	-0.628***	-0.608***	-0.614***	-0.501***	-0.617***	-0.558***
	(0.02)	(0.02)	(0.02)	(0.02)	(0.02)	(0.02)	(0.02)
Angina/Coronary Artery Disease	-0.579***	-0.591***	-0.558***	-0.600***	-0.498***	-0.591***	-0.548***
	(0.05)	(0.05)	(0.05)	(0.04)	(0.04)	(0.05)	(0.04)
Congestive Heart Failure	-0.838***	-0.909***	-0.980***	-0.910***	-0.690***	-0.925***	-0.672***
	(0.07)	(0.07)	(0.07)	(0.07)	(0.06)	(0.07)	(0.06)
Myocardial Infarction Question	-0.443***	-0.445***	-0.469***	-0.439***	-0.383***	-0.463***	-0.371***
	(0.05)	(0.05)	(0.05)	(0.05)	(0.05)	(0.05)	(0.05)
Other Heart Conditions Question	-0.730***	-0.728***	-0.730***	-0.658***	-0.634***	-0.722***	-0.629***
	(0.03)	(0.03)	(0.03)	(0.03)	(0.03)	(0.03)	(0.03)
Stroke	-0.753***	-0.812***	-0.927***	-0.698***	-0.567***	-0.886***	-0.411***
	(0.06)	(0.06)	(0.06)	(0.06)	(0.06)	(0.06)	(0.06)
COPD	-1.388***	-1.463***	-1.481***	-1.529***	-1.304***	-1.503***	-1.329***
	(0.04)	(0.04)	(0.04)	(0.04)	(0.04)	(0.04)	(0.04)
Inflammatory Bowel Disease	-0.348***	-0.324***	-0.325***	-0.292***	-0.334***	-0.295***	-0.282***
	(0.06)	(0.06)	(0.07)	(0.06)	(0.06)	(0.06)	(0.06)
Arthritis of Hip/Knee	-1.096***	-1.087***	-1.125***	-0.848***	-0.683***	-1.081***	-0.678***
	(0.03)	(0.03)	(0.03)	(0.03)	(0.03)	(0.03)	(0.03)
Arthritis of Hand/Wrist	-0.567***	-0.546***	-0.550***	-0.498***	-0.540***	-0.556***	-0.498***
	(0.03)	(0.03)	(0.03)	(0.03)	(0.03)	(0.03)	(0.03)
Osteoporosis	-0.350***	-0.371***	-0.387***	-0.337***	-0.340***	-0.366***	-0.308***
	(0.04)	(0.04)	(0.04)	(0.04)	(0.03)	(0.04)	(0.03)
Sciatica	-0.641***	-0.632***	-0.638***	-0.576***	-0.463***	-0.623***	-0.492***
	(0.03)	(0.03)	(0.03)	(0.03)	(0.03)	(0.03)	(0.03)
Diabetes	-0.619***	-0.639***	-0.668***	-0.569***	-0.507***	-0.649***	-0.472***
	(0.03)	(0.03)	(0.03)	(0.03)	(0.03)	(0.03)	(0.03)
Any Cancer	-0.454***	-0.459***	-0.441***	-0.450***	-0.411***	-0.461***	-0.403***
	(0.03)	(0.03)	(0.03)	(0.03)	(0.03)	(0.03)	(0.03)
Hypertension_Missing	-0.310	-0.326*	-0.318	-0.366*	-0.406**	-0.319	-0.337*
	(0.16)	(0.16)	(0.17)	(0.16)	(0.15)	(0.16)	(0.16)
Angina/Coronary Artery Disease_Missing	-0.0536	-0.110	-0.101	-0.125	-0.139	-0.0934	-0.123
	(0.13)	(0.13)	(0.13)	(0.13)	(0.13)	(0.13)	(0.12)
Congestive Heart Failure_Missing	-0.232	-0.167	-0.163	-0.297*	-0.219	-0.180	-0.300*
	(0.14)	(0.14)	(0.14)	(0.14)	(0.13)	(0.14)	(0.13)
Myocardial Infarction Question_Missing	-0.0373	-0.00857	-0.0936	-0.0246	-0.0281	-0.0764	0.0759
	(0.16)	(0.16)	(0.17)	(0.16)	(0.15)	(0.16)	(0.15)
Other Heart Conditions Question_Missing	-0.263*	-0.233	-0.285*	-0.244	-0.286*	-0.228	-0.241
	(0.13)	(0.13)	(0.14)	(0.13)	(0.13)	(0.13)	(0.12)
Stroke_Missing	-0.120	-0.139	-0.154	-0.151	-0.0876	-0.167	-0.0794
	(0.15)	(0.15)	(0.15)	(0.15)	(0.15)	(0.15)	(0.14)
COPD_Missing	-0.286	-0.355*	-0.303	-0.283	-0.244	-0.297	-0.290
	(0.16)	(0.16)	(0.17)	(0.16)	(0.16)	(0.17)	(0.15)
Inflammatory Bowel Disease_Missing	-0.138	-0.0947	-0.138	-0.139	-0.0980	-0.127	-0.0826
	(0.13)	(0.13)	(0.13)	(0.13)	(0.12)	(0.13)	(0.12)
Arthritis of Hip/Knee_Missing	-0.454***	-0.408**	-0.431**	-0.313*	-0.268*	-0.461***	-0.249*
	(0.13)	(0.13)	(0.13)	(0.13)	(0.12)	(0.13)	(0.12)
Arthritis of Hand/Wrist_Missing	-0.230	-0.256*	-0.253*	-0.221	-0.243*	-0.270*	-0.225*
	(0.12)	(0.12)	(0.13)	(0.12)	(0.11)	(0.13)	(0.11)
Osteoporosis_Missing	-0.128	-0.194	-0.177	-0.0982	-0.127	-0.175	-0.108
	(0.12)	(0.12)	(0.12)	(0.12)	(0.11)	(0.12)	(0.11)
Sciatica_Missing	-0.495***	-0.450***	-0.475***	-0.468***	-0.375**	-0.441**	-0.427**
	(0.13)	(0.13)	(0.14)	(0.14)	(0.13)	(0.14)	(0.13)
Diabetes_Missing	0.122	0.183	0.0992	0.101	0.161	0.108	0.175
	(0.15)	(0.16)	(0.16)	(0.15)	(0.15)	(0.16)	(0.14)
Any Cancer_Missing	-0.158	-0.149	-0.119	-0.110	-0.0972	-0.139	-0.0818
	(0.17)	(0.17)	(0.17)	(0.17)	(0.16)	(0.17)	(0.16)
Housing Category							
Owned or being bought by you	0	0	0	0	0	0	0
	(.)	(.)	(.)	(.)	(.)	(.)	(.)
Owned or being bought by someone in your family	-0.460***	-0.497***	-0.543***	-0.484***	-0.422***	-0.520***	-0.352***
	(0.05)	(0.05)	(0.05)	(0.05)	(0.05)	(0.05)	(0.05)
Rented for money	-0.220***	-0.269***	-0.310***	-0.237***	-0.111**	-0.272***	-0.0617
	(0.04)	(0.04)	(0.04)	(0.04)	(0.04)	(0.04)	(0.04)
Live without payment of rent	-0.440***	-0.444***	-0.519***	-0.414***	-0.441***	-0.514***	-0.344***
	(0.10)	(0.10)	(0.11)	(0.10)	(0.09)	(0.10)	(0.09)
None of the above	-0.112	-0.148*	-0.182**	-0.134*	-0.0528	-0.139*	0.0288
	(0.07)	(0.07)	(0.07)	(0.07)	(0.06)	(0.07)	(0.06)
Missing	-0.137	-0.143	-0.193*	-0.128	-0.0678	-0.157	-0.0422
	(0.09)	(0.09)	(0.09)	(0.09)	(0.09)	(0.09)	(0.08)
BMI Category							
Underweight ( < 20)	0	0	0	0	0	0	0
	(.)	(.)	(.)	(.)	(.)	(.)	(.)
Normal (20-24)	0.288***	0.336***	0.361***	0.422***	0.354***	0.408***	0.272***
	(0.06)	(0.06)	(0.06)	(0.06)	(0.06)	(0.06)	(0.05)
Overweight (25-29)	0.00587	0.0817	0.119*	0.262***	0.200***	0.178**	0.0831
	(0.06)	(0.06)	(0.06)	(0.06)	(0.06)	(0.06)	(0.06)
Obese (30-34)	-0.524***	-0.434***	-0.422***	-0.151*	-0.134*	-0.339***	-0.263***
	(0.06)	(0.06)	(0.06)	(0.06)	(0.06)	(0.06)	(0.06)
Morbid Obesity (>=35)	-1.235***	-1.151***	-1.191***	-0.749***	-0.510***	-1.069***	-0.670***
	(0.08)	(0.08)	(0.08)	(0.07)	(0.07)	(0.08)	(0.07)
Missing	-0.184	-0.108	-0.147	0.0766	0.0856	-0.0361	0.00777
	(0.10)	(0.11)	(0.11)	(0.10)	(0.10)	(0.11)	(0.10)
Current Smoker	-0.713***	-0.770***	-0.733***	-0.826***	-0.589***	-0.762***	-0.636***
	(0.05)	(0.05)	(0.05)	(0.05)	(0.04)	(0.05)	(0.04)
Current Smoker_Missing	0.0580	0.0592	0.0431	0.0256	0.0192	0.0475	-0.0201
	(0.13)	(0.13)	(0.13)	(0.13)	(0.13)	(0.13)	(0.13)
Bone Density Test for Osteoporosis	0.0966**	0.141***	0.153***	0.133***	0.116***	0.153***	0.0716*
	(0.03)	(0.03)	(0.03)	(0.03)	(0.03)	(0.03)	(0.03)
Bone Density Test for Osteoporosis_Missing	-0.193*	-0.166	-0.226*	-0.211*	-0.283**	-0.222*	-0.201*
	(0.10)	(0.10)	(0.10)	(0.09)	(0.09)	(0.10)	(0.09)
Urine Leakage	-0.708***	-0.712***	-0.762***	-0.565***	-0.547***	-0.708***	-0.441***
	(0.03)	(0.03)	(0.03)	(0.03)	(0.02)	(0.03)	(0.02)
Urine Leakage_Missing	-0.557***	-0.540***	-0.591***	-0.505***	-0.438***	-0.572***	-0.365***
	(0.10)	(0.10)	(0.10)	(0.10)	(0.10)	(0.10)	(0.10)
Talked with Doctor About Physical Activities	0.257***	0.285***	0.309***	0.262***	0.216***	0.293***	0.195***
	(0.02)	(0.02)	(0.02)	(0.02)	(0.02)	(0.02)	(0.02)
Talked with Doctor About Physical Activities_Missing	0.133	0.147	0.155*	0.122	0.107	0.142	0.0826
	(0.08)	(0.08)	(0.08)	(0.08)	(0.07)	(0.08)	(0.07)
ADL Items							
Bathing	-10.84***						
	(0.09)						
Dressing		-11.29***					
		(0.10)					
Eating			-9.176***				
			(0.14)				
Getting In/Out of Chairs				-8.983***			
				(0.06)			
Walking					-9.493***		
					(0.05)		
Using the Toilet						-10.67***	
						(0.11)	
ADL Counts							
0							0
							(.)
1							-7.100***
							(0.05)
2							-9.618***
							(0.06)
3							-12.26***
							(0.11)
4							-14.56***
							(0.16)
5							-16.53***
							(0.18)
6							-13.36***
							(0.20)
Benefit Durantion Less than 1 Year	0.159***	0.156***	0.157***	0.143***	0.120***	0.160***	0.127***
	(0.03)	(0.03)	(0.03)	(0.03)	(0.03)	(0.03)	(0.03)
Plan Duration Less than 5 Year	0.0860*	0.0978*	0.0967*	0.0876*	0.0712	0.0845	0.0750
	(0.04)	(0.04)	(0.04)	(0.04)	(0.04)	(0.04)	(0.04)
Year Trend	0.0941***	0.0927***	0.0996***	0.0820***	0.0921***	0.0927***	0.0838***
	(0.01)	(0.01)	(0.01)	(0.01)	(0.01)	(0.01)	(0.01)
Constant	19.27***	18.57***	17.49***	19.63***	21.91***	17.87***	23.09***
	(0.14)	(0.14)	(0.14)	(0.13)	(0.14)	(0.14)	(0.13)

Notes: This is the full results for Panel A of Table 2 with adjustment for covariates listed in Table 1 except baseline MCS, PUD and MUD. **p*<0.1, ***p*<0.05, ****p*<0.01

## Appendix 2

**Table 5:** Full OLS estimates of ADL measures on MCS

	Each ADL						ADL Counts
	(1)	(2)	(3)	(4)	(5)	(6)	(7)
	MCS	MCS	MCS	MCS	MCS	MCS	MCS
N	473282	473282	473282	473282	473282	473282	473282
R-Squared	0.281	0.279	0.269	0.276	0.277	0.273	0.302
MCS_Baseline	0.469***	0.470***	0.473***	0.470***	0.471***	0.473***	0.461***
	(0.00)	(0.00)	(0.00)	(0.00)	(0.00)	(0.00)	(0.00)
Male	0.0491	0.125***	0.0689*	0.0959**	0.0916**	0.0906**	0.143***
	(0.03)	(0.03)	(0.03)	(0.03)	(0.03)	(0.03)	(0.03)
Race Category							
White	0	0	0	0	0	0	0
	(.)	(.)	(.)	(.)	(.)	(.)	(.)
Black	-0.332***	-0.351***	-0.381***	-0.461***	-0.496***	-0.380***	-0.440***
	(0.06)	(0.06)	(0.06)	(0.06)	(0.06)	(0.06)	(0.06)
Other	-0.630***	-0.612***	-0.579***	-0.676***	-0.636***	-0.582***	-0.683***
	(0.14)	(0.14)	(0.14)	(0.13)	(0.14)	(0.13)	(0.14)
Asian	-1.554***	-1.551***	-1.517***	-1.608***	-1.569***	-1.479***	-1.533***
	(0.25)	(0.26)	(0.26)	(0.25)	(0.26)	(0.25)	(0.26)
Hispanic	-1.336***	-1.271***	-1.319***	-1.311***	-1.362***	-1.323***	-1.222***
	(0.13)	(0.13)	(0.13)	(0.13)	(0.13)	(0.13)	(0.13)
American Native	-0.747*	-0.745*	-0.782*	-0.761*	-0.715*	-0.827*	-0.655*
	(0.33)	(0.34)	(0.33)	(0.34)	(0.34)	(0.33)	(0.33)
Unknown	-0.0378	-0.0135	-0.0384	-0.136	-0.188	-0.0831	-0.128
	(0.21)	(0.21)	(0.21)	(0.21)	(0.21)	(0.21)	(0.21)
Education Category							
<High School	0	0	0	0	0	0	0
	(.)	(.)	(.)	(.)	(.)	(.)	(.)
High Schoor or GED	0.888***	0.907***	0.941***	0.949***	0.914***	0.936***	0.840***
	(0.04)	(0.04)	(0.04)	(0.04)	(0.04)	(0.04)	(0.04)
Some College or 2 year degree	1.127***	1.166***	1.189***	1.206***	1.156***	1.196***	1.090***
	(0.04)	(0.04)	(0.04)	(0.04)	(0.04)	(0.04)	(0.04)
Four Year College Degree or More	1.230***	1.274***	1.298***	1.316***	1.238***	1.307***	1.174***
	(0.04)	(0.05)	(0.05)	(0.05)	(0.05)	(0.05)	(0.04)
Missing	0.599***	0.632***	0.677***	0.665***	0.608***	0.669***	0.612***
	(0.13)	(0.13)	(0.13)	(0.13)	(0.13)	(0.13)	(0.13)
Income Category							
<$10,000	0	0	0	0	0	0	0
	(.)	(.)	(.)	(.)	(.)	(.)	(.)
$10,000-$19,999	0.453***	0.486***	0.497***	0.511***	0.515***	0.488***	0.415***
	(0.07)	(0.07)	(0.07)	(0.07)	(0.07)	(0.07)	(0.07)
20000-29999	0.799***	0.841***	0.869***	0.863***	0.852***	0.862***	0.733***
	(0.07)	(0.07)	(0.07)	(0.07)	(0.07)	(0.07)	(0.07)
30000-49999	1.121***	1.178***	1.210***	1.181***	1.158***	1.201***	1.025***
	(0.07)	(0.07)	(0.07)	(0.07)	(0.07)	(0.07)	(0.07)
>50000	1.487***	1.543***	1.595***	1.524***	1.490***	1.576***	1.343***
	(0.07)	(0.07)	(0.07)	(0.07)	(0.07)	(0.07)	(0.07)
Don't Know	0.756***	0.787***	0.785***	0.757***	0.755***	0.799***	0.681***
	(0.07)	(0.07)	(0.07)	(0.07)	(0.07)	(0.07)	(0.07)
Missing	1.254***	1.300***	1.330***	1.306***	1.292***	1.335***	1.166***
	(0.07)	(0.07)	(0.07)	(0.07)	(0.07)	(0.07)	(0.07)
Marital Status							
Married	0	0	0	0	0	0	0
	(.)	(.)	(.)	(.)	(.)	(.)	(.)
Divorced	0.238***	0.219***	0.280***	0.246***	0.246***	0.261***	0.189***
	(0.04)	(0.04)	(0.04)	(0.04)	(0.04)	(0.04)	(0.04)
Separated	-0.0561	-0.0302	0.0317	-0.00125	0.00732	0.00686	-0.0312
	(0.14)	(0.14)	(0.14)	(0.14)	(0.14)	(0.14)	(0.14)
Widowed	0.657***	0.591***	0.599***	0.641***	0.698***	0.612***	0.689***
	(0.03)	(0.03)	(0.03)	(0.03)	(0.03)	(0.03)	(0.03)
Never Married	0.253***	0.201**	0.260***	0.245***	0.234***	0.250***	0.210**
	(0.07)	(0.07)	(0.07)	(0.07)	(0.07)	(0.07)	(0.07)
Missing	0.622***	0.558***	0.623***	0.673***	0.675***	0.607***	0.606***
	(0.14)	(0.14)	(0.14)	(0.14)	(0.14)	(0.14)	(0.14)
Medical History							
Hypertension	-0.0462*	-0.0577*	-0.0595*	-0.0335	0.0355	-0.0587*	-0.00416
	(0.02)	(0.02)	(0.02)	(0.02)	(0.02)	(0.02)	(0.02)
Angina/Coronary Artery Disease	-0.252***	-0.271***	-0.262***	-0.257***	-0.183***	-0.284***	-0.212***
	(0.05)	(0.05)	(0.05)	(0.05)	(0.05)	(0.05)	(0.05)
Congestive Heart Failure	-0.458***	-0.521***	-0.587***	-0.517***	-0.395***	-0.543***	-0.326***
	(0.07)	(0.07)	(0.07)	(0.07)	(0.07)	(0.07)	(0.07)
Myocardial Infarction Question	-0.0771	-0.0833	-0.111*	-0.0764	-0.0423	-0.104*	-0.0249
	(0.05)	(0.05)	(0.05)	(0.05)	(0.05)	(0.05)	(0.05)
Other Heart Conditions Question	-0.161***	-0.169***	-0.191***	-0.111***	-0.0870**	-0.175***	-0.0829**
	(0.03)	(0.03)	(0.03)	(0.03)	(0.03)	(0.03)	(0.03)
Stroke	-0.427***	-0.472***	-0.547***	-0.425***	-0.382***	-0.532***	-0.235***
	(0.06)	(0.06)	(0.06)	(0.06)	(0.06)	(0.06)	(0.06)
COPD	-0.309***	-0.382***	-0.431***	-0.395***	-0.241***	-0.437***	-0.243***
	(0.04)	(0.04)	(0.04)	(0.04)	(0.04)	(0.04)	(0.04)
Inflammatory Bowel Disease	-0.870***	-0.856***	-0.853***	-0.831***	-0.839***	-0.833***	-0.807***
	(0.07)	(0.07)	(0.07)	(0.07)	(0.07)	(0.07)	(0.07)
Arthritis of Hip/Knee	-0.102***	-0.113***	-0.177***	0.0510	0.130***	-0.126***	0.140***
	(0.03)	(0.03)	(0.03)	(0.03)	(0.03)	(0.03)	(0.03)
Arthritis of Hand/Wrist	-0.340***	-0.333***	-0.343***	-0.294***	-0.302***	-0.348***	-0.269***
	(0.03)	(0.03)	(0.03)	(0.03)	(0.03)	(0.03)	(0.03)
Osteoporosis	-0.273***	-0.294***	-0.319***	-0.266***	-0.256***	-0.297***	-0.231***
	(0.04)	(0.04)	(0.04)	(0.04)	(0.04)	(0.04)	(0.04)
Sciatica	-0.432***	-0.437***	-0.462***	-0.384***	-0.311***	-0.441***	-0.318***
	(0.03)	(0.03)	(0.03)	(0.03)	(0.03)	(0.03)	(0.03)
Diabetes	-0.313***	-0.332***	-0.362***	-0.287***	-0.256***	-0.346***	-0.225***
	(0.03)	(0.03)	(0.03)	(0.03)	(0.03)	(0.03)	(0.03)
Any Cancer	-0.0151	-0.0238	-0.0126	-0.0111	0.0172	-0.0318	0.0268
	(0.03)	(0.03)	(0.03)	(0.03)	(0.03)	(0.03)	(0.03)
Hypertension_Missing	-0.0542	-0.0668	-0.0558	-0.0909	-0.101	-0.0621	-0.0643
	(0.16)	(0.16)	(0.16)	(0.16)	(0.16)	(0.16)	(0.15)
Angina/Coronary Artery Disease_Missing	-0.138	-0.184	-0.182	-0.185	-0.180	-0.174	-0.159
	(0.14)	(0.14)	(0.14)	(0.14)	(0.14)	(0.13)	(0.13)
Congestive Heart Failure_Missing	0.110	0.158	0.162	0.0801	0.134	0.146	0.101
	(0.14)	(0.14)	(0.14)	(0.14)	(0.14)	(0.14)	(0.14)
Myocardial Infarction Question_Missing	0.0627	0.0874	0.0315	0.0584	0.0456	0.0374	0.115
	(0.16)	(0.17)	(0.16)	(0.16)	(0.16)	(0.16)	(0.16)
Other Heart Conditions Question_Missing	-0.369**	-0.350*	-0.412**	-0.352**	-0.362**	-0.349*	-0.357**
	(0.14)	(0.14)	(0.14)	(0.13)	(0.13)	(0.14)	(0.13)
Stroke_Missing	-0.328	-0.336	-0.326	-0.359*	-0.341	-0.353	-0.307
	(0.18)	(0.18)	(0.18)	(0.18)	(0.18)	(0.18)	(0.18)
COPD_Missing	0.0833	0.0297	0.0664	0.0850	0.105	0.0739	0.0820
	(0.18)	(0.18)	(0.18)	(0.18)	(0.18)	(0.18)	(0.18)
Inflammatory Bowel Disease_Missing	-0.441**	-0.407**	-0.439**	-0.440**	-0.419**	-0.429**	-0.429**
	(0.14)	(0.14)	(0.14)	(0.14)	(0.13)	(0.14)	(0.14)
Arthritis of Hip/Knee_Missing	-0.349*	-0.322*	-0.350*	-0.260	-0.237	-0.375**	-0.216
	(0.14)	(0.14)	(0.14)	(0.14)	(0.14)	(0.14)	(0.14)
Arthritis of Hand/Wrist_Missing	0.00572	-0.0161	-0.0130	0.00754	-0.00123	-0.0300	0.0255
	(0.12)	(0.12)	(0.12)	(0.12)	(0.12)	(0.13)	(0.12)
Osteoporosis_Missing	-0.344*	-0.395**	-0.387**	-0.329*	-0.346*	-0.381**	-0.339**
	(0.13)	(0.13)	(0.14)	(0.13)	(0.13)	(0.13)	(0.13)
Sciatica_Missing	-0.176	-0.147	-0.179	-0.151	-0.0944	-0.143	-0.125
	(0.14)	(0.14)	(0.14)	(0.14)	(0.14)	(0.14)	(0.13)
Diabetes_Missing	-0.114	-0.0676	-0.143	-0.125	-0.0910	-0.128	-0.110
	(0.16)	(0.16)	(0.16)	(0.16)	(0.16)	(0.16)	(0.16)
Any Cancer_Missing	-0.273	-0.263	-0.225	-0.242	-0.246	-0.250	-0.260
	(0.17)	(0.17)	(0.17)	(0.17)	(0.17)	(0.17)	(0.17)
Housing Category							
Owned or being bought by you	0	0	0	0	0	0	0
	(.)	(.)	(.)	(.)	(.)	(.)	(.)
Owned or being bought by someone in your family	-0.178**	-0.207***	-0.236***	-0.207***	-0.180***	-0.225***	-0.115*
	(0.05)	(0.05)	(0.05)	(0.05)	(0.05)	(0.05)	(0.05)
Rented for money	-0.361***	-0.395***	-0.417***	-0.386***	-0.337***	-0.394***	-0.292***
	(0.05)	(0.05)	(0.05)	(0.05)	(0.05)	(0.05)	(0.05)
Live without payment of rent	-0.268**	-0.270*	-0.324**	-0.266*	-0.288**	-0.326**	-0.214*
	(0.10)	(0.10)	(0.10)	(0.10)	(0.10)	(0.10)	(0.10)
None of the above	-0.682***	-0.705***	-0.718***	-0.708***	-0.679***	-0.692***	-0.632***
	(0.07)	(0.07)	(0.07)	(0.07)	(0.07)	(0.07)	(0.07)
Missing	-0.201*	-0.206*	-0.247*	-0.202*	-0.174	-0.217*	-0.142
	(0.09)	(0.10)	(0.10)	(0.10)	(0.10)	(0.10)	(0.10)
BMI Category							
Underweight ( < 20)	0	0	0	0	0	0	0
	(.)	(.)	(.)	(.)	(.)	(.)	(.)
Normal (20-24)	0.223***	0.256***	0.246***	0.324***	0.291***	0.309***	0.208***
	(0.06)	(0.06)	(0.06)	(0.06)	(0.06)	(0.06)	(0.06)
Overweight (25-29)	0.421***	0.469***	0.454***	0.605***	0.573***	0.537***	0.459***
	(0.06)	(0.06)	(0.06)	(0.06)	(0.06)	(0.06)	(0.06)
Obese (30-34)	0.545***	0.598***	0.546***	0.803***	0.805***	0.659***	0.686***
	(0.06)	(0.06)	(0.06)	(0.06)	(0.06)	(0.06)	(0.06)
Morbid Obesity (>=35)	0.724***	0.765***	0.653***	1.045***	1.146***	0.807***	1.015***
	(0.08)	(0.08)	(0.08)	(0.08)	(0.08)	(0.08)	(0.08)
Missing	0.155	0.206	0.140	0.328**	0.319**	0.259*	0.270*
	(0.11)	(0.11)	(0.11)	(0.11)	(0.11)	(0.11)	(0.11)
Current Smoker	-0.661***	-0.705***	-0.668***	-0.732***	-0.607***	-0.699***	-0.645***
	(0.04)	(0.04)	(0.04)	(0.04)	(0.04)	(0.04)	(0.04)
Current Smoker_Missing	0.0593	0.0597	0.0393	0.0404	0.0419	0.0488	0.0317
	(0.14)	(0.14)	(0.14)	(0.14)	(0.14)	(0.14)	(0.13)
Bone Density Test for Osteoporosis	0.193***	0.226***	0.230***	0.224***	0.218***	0.235***	0.186***
	(0.03)	(0.03)	(0.03)	(0.03)	(0.03)	(0.03)	(0.03)
Bone Density Test for Osteoporosis_Missing	-0.261**	-0.240*	-0.282**	-0.278**	-0.313**	-0.282**	-0.262**
	(0.10)	(0.10)	(0.10)	(0.10)	(0.10)	(0.10)	(0.09)
Urine Leakage	-0.589***	-0.594***	-0.636***	-0.509***	-0.513***	-0.592***	-0.460***
	(0.03)	(0.03)	(0.03)	(0.03)	(0.03)	(0.03)	(0.03)
Urine Leakage_Missing	-0.513***	-0.497***	-0.518***	-0.490***	-0.472***	-0.517***	-0.423***
	(0.11)	(0.11)	(0.11)	(0.11)	(0.10)	(0.11)	(0.10)
Talked with Doctor About Physical Activities	0.155***	0.175***	0.194***	0.164***	0.147***	0.181***	0.132***
	(0.02)	(0.03)	(0.02)	(0.02)	(0.02)	(0.02)	(0.02)
Talked with Doctor About Physical Activities_Missing	0.0273	0.0383	0.0442	0.0251	0.0212	0.0348	0.00178
	(0.08)	(0.08)	(0.08)	(0.08)	(0.08)	(0.08)	(0.08)
ADL Items							
Bathing	-7.931***						
	(0.09)						
Dressing		-8.718***					
		(0.10)					
Eating			-10.13***				
			(0.15)				
Getting In/Out of Chairs				-5.340***			
				(0.06)			
Walking					-4.368***		
					(0.05)		
Using the Toilet						-8.992***	
						(0.11)	
ADL Scale							
0							0
							(.)
1							-2.293***
							(0.05)
2							-4.278***
							(0.07)
3							-6.707***
							(0.13)
4							-9.397***
							(0.17)
5							-11.80***
							(0.22)
6							-13.36***
							(0.22)
Benefit Duration Less than 1 Year	-0.0816**	-0.0810**	-0.0750**	-0.0930***	-0.107***	-0.0746**	-0.104***
	(0.03)	(0.03)	(0.03)	(0.03)	(0.03)	(0.03)	(0.03)
Plan Duration Less than 5 Year	0.00172	0.0116	0.0144	0.00296	-0.00632	0.00208	-0.00469
	(0.04)	(0.04)	(0.04)	(0.04)	(0.04)	(0.04)	(0.04)
Year Trend	0.158***	0.157***	0.161***	0.152***	0.159***	0.156***	0.148***
	(0.01)	(0.01)	(0.01)	(0.01)	(0.01)	(0.01)	(0.01)
Constant	26.71***	26.46***	26.60***	26.66***	26.43***	27.65***	
	(0.16)	(0.16)	(0.15)	(0.16)	(0.16)	(0.15)	

Notes: This is the full results for Panel B of Table 2 with adjustment for covariates listed in Table 1 except baseline PCS, PUD and MUD. **p*<0.1, ***p*<0.05, ****p*<0.01

## Appendix 3

**Table 6:** Full Results for Marginal Effects of On-set ADLs on Physical Unhealthy Days by using Zero-inflated Negative Binomial Regression

	Each ADL						ADL Counts
	(1)	(2)	(3)	(4)	(5)	(6)	(7)
	PUD	PUD	PUD	PUD	PUD	PUD	PUD
N	473282	473282	473282	473282	473282	473282	473282
Physically Unhealthy Days (PHYUD)_Baseline	0.446***	0.478***	0.590***	0.382***	0.264***	0.529***	0.171***
	(0.03)	(0.03)	(0.03)	(0.02)	(0.04)	(0.03)	(0.00)
Mentally Unhealthy Days (MENUD)_Baseline							
Male	0.184***	0.118***	0.181***	0.144***	0.129***	0.153***	0.111***
	(0.03)	(0.03)	(0.03)	(0.03)	(0.03)	(0.03)	(0.03)
Race Category							
White	0	0	0	0	0	0	0
	(.)	(.)	(.)	(.)	(.)	(.)	(.)
Black	0.246***	0.259***	0.258***	0.350***	0.446***	0.269***	0.393***
	(0.05)	(0.05)	(0.05)	(0.05)	(0.05)	(0.05)	(0.05)
Other	-0.165	-0.166	-0.206*	-0.0760	-0.136	-0.195*	-0.0743
	(0.09)	(0.09)	(0.09)	(0.10)	(0.09)	(0.09)	(0.09)
Asian	-0.135	-0.128	-0.132	-0.0922	-0.130	-0.188*	-0.155
	(0.08)	(0.08)	(0.08)	(0.09)	(0.08)	(0.08)	(0.08)
Hispanic	0.482***	0.436***	0.463***	0.444***	0.498***	0.463***	0.422***
	(0.08)	(0.08)	(0.08)	(0.08)	(0.08)	(0.08)	(0.08)
American Native	0.628*	0.643*	0.687*	0.725**	0.531*	0.816**	0.391
	(0.26)	(0.26)	(0.27)	(0.27)	(0.24)	(0.29)	(0.21)
Unknown	-0.544*	-0.599**	-0.563*	-0.490*	-0.423*	-0.523*	-0.433*
	(0.23)	(0.21)	(0.22)	(0.22)	(0.21)	(0.23)	(0.21)
Education Category							
<High School	0	0	0	0	0	0	0
	(.)	(.)	(.)	(.)	(.)	(.)	(.)
High School or GED	-0.441***	-0.465***	-0.505***	-0.474***	-0.445***	-0.481***	-0.398***
	(0.04)	(0.04)	(0.04)	(0.04)	(0.04)	(0.04)	(0.04)
Some College or 2 year degree	-0.522***	-0.563***	-0.584***	-0.585***	-0.558***	-0.576***	-0.543***
	(0.04)	(0.04)	(0.04)	(0.04)	(0.04)	(0.04)	(0.04)
Four Year College Degree or More	-0.857***	-0.898***	-0.910***	-0.903***	-0.868***	-0.907***	-0.873***
	(0.04)	(0.04)	(0.04)	(0.04)	(0.04)	(0.04)	(0.04)
Missing	-0.257*	-0.319*	-0.334**	-0.335**	-0.283*	-0.326*	-0.247
	(0.13)	(0.13)	(0.13)	(0.12)	(0.13)	(0.13)	(0.13)
Income Category							
<$10,000	0	0	0	0	0	0	0
	(.)	(.)	(.)	(.)	(.)	(.)	(.)
$10,000-$19,999	-0.289***	-0.304***	-0.315***	-0.341***	-0.323***	-0.302***	-0.281***
	(0.05)	(0.05)	(0.05)	(0.05)	(0.05)	(0.05)	(0.04)
20000-29999	-0.437***	-0.457***	-0.484***	-0.472***	-0.450***	-0.467***	-0.386***
	(0.05)	(0.05)	(0.05)	(0.05)	(0.05)	(0.05)	(0.05)
30000-49999	-0.649***	-0.700***	-0.730***	-0.701***	-0.644***	-0.718***	-0.561***
	(0.05)	(0.05)	(0.05)	(0.05)	(0.05)	(0.05)	(0.05)
>50000	-0.772***	-0.826***	-0.887***	-0.793***	-0.718***	-0.858***	-0.611***
	(0.06)	(0.05)	(0.06)	(0.06)	(0.06)	(0.06)	(0.05)
Don't Know	-0.262***	-0.288***	-0.286***	-0.271***	-0.232***	-0.298***	-0.180**
	(0.06)	(0.06)	(0.06)	(0.06)	(0.06)	(0.06)	(0.06)
Missing	-0.486***	-0.518***	-0.560***	-0.531***	-0.492***	-0.556***	-0.390***
	(0.06)	(0.06)	(0.06)	(0.06)	(0.06)	(0.06)	(0.06)
Marital Status							
Married	0	0	0	0	0	0	0
	(.)	(.)	(.)	(.)	(.)	(.)	(.)
Divorced	-0.0204	-0.00176	-0.0745*	-0.0293	-0.0152	-0.0493	0.00959
	(0.04)	(0.04)	(0.04)	(0.04)	(0.04)	(0.04)	(0.04)
Separated	-0.0975	-0.114	-0.172	-0.149	-0.122	-0.149	-0.0916
	(0.10)	(0.10)	(0.10)	(0.10)	(0.11)	(0.10)	(0.11)
Widowed	-0.122***	-0.0650*	-0.0753*	-0.105**	-0.159***	-0.0902**	-0.145***
	(0.03)	(0.03)	(0.03)	(0.03)	(0.03)	(0.03)	(0.03)
Never Married	-0.339***	-0.287***	-0.353***	-0.335***	-0.344***	-0.341***	-0.316***
	(0.06)	(0.06)	(0.06)	(0.06)	(0.06)	(0.06)	(0.06)
Missing	-0.290*	-0.203	-0.281*	-0.323**	-0.360**	-0.269*	-0.327**
	(0.13)	(0.13)	(0.12)	(0.12)	(0.13)	(0.13)	(0.13)
Medical History							
Hypertension	0.102***	0.119***	0.112***	0.0846**	-0.00776	0.114***	0.0227
	(0.03)	(0.03)	(0.03)	(0.03)	(0.03)	(0.03)	(0.03)
Angina/Coronary Artery Disease	0.243***	0.252***	0.240***	0.254***	0.182***	0.272***	0.185***
	(0.04)	(0.04)	(0.04)	(0.04)	(0.04)	(0.04)	(0.04)
Congestive Heart Failure	0.288***	0.328***	0.376***	0.306***	0.230***	0.338***	0.197***
	(0.05)	(0.05)	(0.05)	(0.05)	(0.05)	(0.05)	(0.04)
Myocardial Infarction Question	0.0983*	0.126**	0.143**	0.0917*	0.0533	0.134**	0.0416
	(0.05)	(0.05)	(0.05)	(0.05)	(0.04)	(0.05)	(0.04)
Other Heart Conditions Question	0.227***	0.234***	0.249***	0.185***	0.129***	0.238***	0.110***
	(0.03)	(0.03)	(0.03)	(0.03)	(0.03)	(0.03)	(0.03)
Stroke	0.281***	0.310***	0.376***	0.260***	0.188***	0.353***	0.117*
	(0.05)	(0.05)	(0.05)	(0.05)	(0.05)	(0.05)	(0.05)
COPD	0.556***	0.616***	0.649***	0.620***	0.456***	0.656***	0.424***
	(0.03)	(0.03)	(0.03)	(0.03)	(0.03)	(0.03)	(0.03)
Inflammatory Bowel Disease	0.284***	0.274***	0.271***	0.254***	0.251***	0.267***	0.194***
	(0.05)	(0.05)	(0.05)	(0.05)	(0.05)	(0.05)	(0.04)
Arthritis of Hip/Knee	0.332***	0.339***	0.393***	0.186***	0.0799**	0.344***	0.0636*
	(0.03)	(0.03)	(0.03)	(0.03)	(0.03)	(0.03)	(0.03)
Arthritis of Hand/Wrist	0.267***	0.264***	0.270***	0.219***	0.214***	0.275***	0.155***
	(0.03)	(0.03)	(0.03)	(0.03)	(0.03)	(0.03)	(0.02)
Osteoporosis	0.201***	0.213***	0.244***	0.191***	0.173***	0.219***	0.141***
	(0.03)	(0.03)	(0.03)	(0.03)	(0.03)	(0.03)	(0.03)
Sciatica	0.418***	0.427***	0.435***	0.360***	0.278***	0.423***	0.261***
	(0.03)	(0.03)	(0.03)	(0.03)	(0.03)	(0.03)	(0.03)
Diabetes	0.211***	0.224***	0.256***	0.177***	0.143***	0.237***	0.104***
	(0.03)	(0.03)	(0.03)	(0.03)	(0.03)	(0.03)	(0.03)
Any Cancer	0.167***	0.177***	0.170***	0.168***	0.130***	0.182***	0.123***
	(0.03)	(0.03)	(0.03)	(0.03)	(0.03)	(0.03)	(0.03)
Hypertension_Missing	0.0723	0.116	0.117	0.104	0.105	0.0900	0.0282
	(0.18)	(0.18)	(0.18)	(0.18)	(0.18)	(0.18)	(0.18)
Angina/Coronary Artery Disease_Missing	-0.0257	0.00380	-0.0276	0.0377	0.0969	0.00967	0.0849
	(0.11)	(0.11)	(0.11)	(0.11)	(0.11)	(0.11)	(0.11)
Congestive Heart Failure_Missing	0.128	0.0757	0.0651	0.173	0.0784	0.0682	0.127
	(0.13)	(0.13)	(0.13)	(0.13)	(0.13)	(0.13)	(0.12)
Myocardial Infarction Question_Missing	-0.107	-0.139	-0.0832	-0.141	-0.0945	-0.102	-0.124
	(0.13)	(0.13)	(0.12)	(0.13)	(0.13)	(0.13)	(0.13)
Other Heart Conditions Question_Missing	0.0163	0.00515	0.0355	0.00732	0.0506	-0.00373	0.0721
	(0.12)	(0.12)	(0.12)	(0.12)	(0.13)	(0.13)	(0.12)
Stroke_Missing	-0.0651	-0.0371	-0.0155	-0.0125	-0.0801	0.000915	-0.0926
	(0.14)	(0.13)	(0.13)	(0.13)	(0.13)	(0.14)	(0.13)
COPD_Missing	0.0924	0.120	0.111	0.0566	0.0354	0.136	-0.00506
	(0.13)	(0.13)	(0.13)	(0.13)	(0.14)	(0.13)	(0.13)
Inflammatory Bowel Disease_Missing	0.426***	0.381***	0.367***	0.399***	0.357**	0.374***	0.355**
	(0.11)	(0.11)	(0.11)	(0.11)	(0.11)	(0.11)	(0.11)
Arthritis of Hip/Knee_Missing	0.220	0.181	0.211	0.0793	0.0206	0.212	0.00899
	(0.12)	(0.12)	(0.12)	(0.12)	(0.12)	(0.12)	(0.12)
Arthritis of Hand/Wrist_Missing	0.195	0.240	0.216	0.210	0.213	0.251*	0.177
	(0.12)	(0.12)	(0.12)	(0.12)	(0.12)	(0.12)	(0.12)
Osteoporosis_Missing	0.199	0.232*	0.205	0.152	0.191	0.198	0.190
	(0.11)	(0.12)	(0.11)	(0.11)	(0.12)	(0.11)	(0.12)
Sciatica_Missing	0.302*	0.259*	0.293*	0.265*	0.277*	0.283*	0.278*
	(0.13)	(0.13)	(0.13)	(0.13)	(0.13)	(0.13)	(0.13)
Diabetes_Missing	0.0459	0.0140	0.0818	0.0818	0.139	0.0409	0.110
	(0.16)	(0.16)	(0.16)	(0.16)	(0.17)	(0.16)	(0.17)
Any Cancer_Missing	-0.0762	-0.0841	-0.123	-0.128	-0.142	-0.143	-0.167
	(0.15)	(0.15)	(0.15)	(0.15)	(0.15)	(0.15)	(0.15)
Housing Category							
Owned or being bought by you	0	0	0	0	0	0	0
	(.)	(.)	(.)	(.)	(.)	(.)	(.)
Owned or being bought by someone in your family	0.0775	0.110*	0.155**	0.111*	0.0718	0.135*	0.0240
	(0.06)	(0.06)	(0.06)	(0.05)	(0.06)	(0.06)	(0.05)
Rented for money	0.136***	0.160***	0.192***	0.146***	0.0808*	0.167***	0.0507
	(0.04)	(0.04)	(0.04)	(0.04)	(0.04)	(0.04)	(0.04)
Live without payment of rent	0.214*	0.216*	0.274**	0.209*	0.233*	0.284**	0.168
	(0.10)	(0.10)	(0.10)	(0.10)	(0.10)	(0.10)	(0.09)
None of the above	0.309***	0.313***	0.345***	0.324***	0.284***	0.303***	0.250***
	(0.07)	(0.07)	(0.07)	(0.07)	(0.07)	(0.07)	(0.06)
Missing	0.240*	0.234*	0.302**	0.217*	0.184	0.265**	0.140
	(0.10)	(0.10)	(0.10)	(0.10)	(0.10)	(0.10)	(0.09)
BMI Category							
Underweight ( < 20)	0	0	0	0	0	0	0
	(.)	(.)	(.)	(.)	(.)	(.)	(.)
Normal (20-24)	-0.210***	-0.258***	-0.264***	-0.297***	-0.221***	-0.312***	-0.157**
	(0.06)	(0.06)	(0.06)	(0.06)	(0.06)	(0.06)	(0.06)
Overweight (25-29)	-0.198**	-0.267***	-0.271***	-0.379***	-0.319***	-0.329***	-0.234***
	(0.06)	(0.06)	(0.06)	(0.06)	(0.06)	(0.07)	(0.06)
Obese (30-34)	-0.0438	-0.116	-0.0842	-0.288***	-0.295***	-0.176**	-0.199**
	(0.07)	(0.07)	(0.07)	(0.07)	(0.07)	(0.07)	(0.06)
Morbid Obesity (>=35)	-0.0230	-0.0841	0.000555	-0.296***	-0.409***	-0.122	-0.300***
	(0.07)	(0.08)	(0.08)	(0.08)	(0.07)	(0.08)	(0.07)
Missing	0.0611	0.00821	0.0534	-0.102	-0.0334	-0.0572	0.0130
	(0.12)	(0.12)	(0.12)	(0.12)	(0.12)	(0.12)	(0.12)
Current Smoker	0.445***	0.483***	0.472***	0.488***	0.339***	0.488***	0.322***
	(0.04)	(0.04)	(0.04)	(0.04)	(0.04)	(0.04)	(0.04)
Current Smoker_Missing	-0.212	-0.247	-0.248	-0.236	-0.263	-0.222	-0.235
	(0.15)	(0.14)	(0.14)	(0.14)	(0.14)	(0.14)	(0.14)
Bone Density Test for Osteoporosis	-0.00609	-0.0320	-0.0484	-0.0267	-0.0236	-0.0410	0.00821
	(0.03)	(0.03)	(0.03)	(0.03)	(0.03)	(0.03)	(0.03)
Bone Density Test for Osteoporosis_Missing	0.184*	0.142	0.180*	0.196*	0.214*	0.196*	0.173*
	(0.09)	(0.09)	(0.08)	(0.09)	(0.08)	(0.09)	(0.08)
Urine Leakage	0.139***	0.146***	0.193***	0.0551*	0.0306	0.148***	-0.0442
	(0.03)	(0.03)	(0.03)	(0.02)	(0.03)	(0.03)	(0.02)
Urine Leakage_Missing	0.314**	0.314**	0.353***	0.320**	0.299**	0.342**	0.252*
	(0.11)	(0.11)	(0.10)	(0.11)	(0.11)	(0.11)	(0.11)
Talked with Doctor About Physical Activities	-0.124***	-0.142***	-0.159***	-0.135***	-0.120***	-0.146***	-0.122***
	(0.03)	(0.03)	(0.03)	(0.03)	(0.03)	(0.03)	(0.02)
Talked with Doctor About Physical Activities_Missing	0.137	0.133	0.146	0.138	0.167	0.133	0.177
	(0.09)	(0.09)	(0.09)	(0.09)	(0.09)	(0.09)	(0.09)
ADL Items							
Bathing	6.239***						
	(0.06)						
Dressing		6.828***					
		(0.09)					
Eating			6.341***				
			(0.12)				
Getting In/Out of Chairs				4.929***			
				(0.04)			
Walking					4.960***		
					(0.04)		
Using the Toilet						6.715***	
						(0.10)	
ADL Scale							
0							0
							(.)
1							4.355***
							(0.06)
2							7.261***
							(0.08)
3							10.59***
							(0.16)
4							13.88***
							(0.23)
5							16.35***
							(0.25)
6							14.22***
							(0.25)
Benefit Durantion Less than 1 Year	-0.105***	-0.107***	-0.119***	-0.101***	-0.0747**	-0.113***	-0.0817**
	(0.03)	(0.03)	(0.03)	(0.03)	(0.03)	(0.03)	(0.03)
Plan Duration Less than 5 Year	-0.0675	-0.0770*	-0.0825*	-0.0691*	-0.0539	-0.0747*	-0.0456
	(0.03)	(0.03)	(0.03)	(0.03)	(0.03)	(0.03)	(0.03)
Year Trend	-0.0753***	-0.0737***	-0.0772***	-0.0709***	-0.0807***	-0.0732***	-0.0793***
	(0.01)	(0.01)	(0.01)	(0.01)	(0.01)	(0.01)	(0.01)

Notes: This is the full results for Panel A of Table 3 with adjustment for covariates listed in Table 1 except baseline PCS, MCS and MUD. **p*<0.1, ***p*<0.05, ****p*<0.01

## Appendix 4

**Table 7:** Full Results for Marginal Effects of On-set ADLs on Mental Unhealthy Days by using Zero-inflated Negative Binomial Regression

	Each ADL						ADL Counts
	(1)	(2)	(3)	(4)	(5)	(6)	(7)
	MENUD	MENUD	MENUD	MENUD	MENUD	MENUD	MENUD
N	473282	473282	473282	473282	473282	473282	473282
Physically Unhealthy Days (PHYUD)_Baseline							
Mentally Unhealthy Days (MENUD)_Baseline	0.933***	0.935***	0.961***	0.942***	0.958***	0.946***	0.883***
	(0.02)	(0.02)	(0.02)	(0.02)	(0.02)	(0.02)	(0.02)
Male	-0.153***	-0.183***	-0.138***	-0.173***	-0.180***	-0.164***	-0.207***
	(0.03)	(0.03)	(0.03)	(0.03)	(0.03)	(0.03)	(0.03)
Race Category							
White	0	0	0	0	0	0	0
	(.)	(.)	(.)	(.)	(.)	(.)	(.)
Black	0.212***	0.217***	0.241***	0.275***	0.300***	0.229***	0.270***
	(0.04)	(0.04)	(0.04)	(0.04)	(0.04)	(0.04)	(0.05)
Other	-0.0592	-0.0596	-0.0903	-0.0322	-0.0778	-0.0920	-0.0477
	(0.07)	(0.07)	(0.07)	(0.07)	(0.07)	(0.07)	(0.07)
Asian	-0.0604	-0.0703	-0.0775	-0.0405	-0.0598	-0.107	-0.0664
	(0.07)	(0.07)	(0.07)	(0.07)	(0.07)	(0.07)	(0.07)
Hispanic	0.304***	0.282***	0.314***	0.285***	0.302***	0.306***	0.261***
	(0.07)	(0.07)	(0.07)	(0.07)	(0.07)	(0.07)	(0.07)
American Native	0.517*	0.490*	0.476*	0.461*	0.437*	0.531*	0.420*
	(0.21)	(0.20)	(0.20)	(0.20)	(0.20)	(0.21)	(0.20)
Unknown	-0.388	-0.389	-0.402*	-0.365	-0.291	-0.398*	-0.329
	(0.21)	(0.20)	(0.20)	(0.21)	(0.22)	(0.20)	(0.21)
Education Category							
<High School	0	0	0	0	0	0	0
	(.)	(.)	(.)	(.)	(.)	(.)	(.)
High School or GED	-0.389***	-0.401***	-0.422***	-0.425***	-0.397***	-0.415***	-0.362***
	(0.03)	(0.03)	(0.03)	(0.03)	(0.03)	(0.03)	(0.03)
Some College or 2 year degree	-0.498***	-0.521***	-0.541***	-0.544***	-0.503***	-0.539***	-0.473***
	(0.03)	(0.03)	(0.03)	(0.03)	(0.04)	(0.03)	(0.04)
Four Year College Degree or More	-0.662***	-0.684***	-0.707***	-0.707***	-0.656***	-0.704***	-0.624***
	(0.04)	(0.04)	(0.04)	(0.04)	(0.04)	(0.04)	(0.04)
Missing	-0.300**	-0.328**	-0.382***	-0.362**	-0.352**	-0.366**	-0.327**
	(0.11)	(0.11)	(0.11)	(0.11)	(0.11)	(0.11)	(0.12)
Income Category							
<$10,000	0	0	0	0	0	0	0
	(.)	(.)	(.)	(.)	(.)	(.)	(.)
$10,000-$19,999	-0.119**	-0.129**	-0.136***	-0.153***	-0.133**	-0.134***	-0.0966*
	(0.04)	(0.04)	(0.04)	(0.04)	(0.04)	(0.04)	(0.04)
20000-29999	-0.247***	-0.267***	-0.277***	-0.278***	-0.271***	-0.275***	-0.221***
	(0.04)	(0.04)	(0.04)	(0.04)	(0.04)	(0.04)	(0.04)
30000-49999	-0.385***	-0.410***	-0.427***	-0.421***	-0.394***	-0.431***	-0.339***
	(0.04)	(0.04)	(0.04)	(0.04)	(0.04)	(0.04)	(0.04)
>50000	-0.573***	-0.599***	-0.623***	-0.592***	-0.557***	-0.617***	-0.503***
	(0.05)	(0.05)	(0.05)	(0.05)	(0.05)	(0.05)	(0.05)
Don't Know	-0.134**	-0.144**	-0.139**	-0.130*	-0.120*	-0.153**	-0.0978
	(0.05)	(0.05)	(0.05)	(0.05)	(0.05)	(0.05)	(0.05)
Missing	-0.393***	-0.408***	-0.418***	-0.418***	-0.408***	-0.428***	-0.352***
	(0.05)	(0.05)	(0.05)	(0.05)	(0.05)	(0.05)	(0.05)
Marital Status							
Married	0	0	0	0	0	0	0
	(.)	(.)	(.)	(.)	(.)	(.)	(.)
Divorced	-0.0646*	-0.0536	-0.0999**	-0.0752*	-0.0650*	-0.0873**	-0.0324
	(0.03)	(0.03)	(0.03)	(0.03)	(0.03)	(0.03)	(0.03)
Separated	0.0976	0.0729	0.0349	0.0719	0.106	0.0597	0.135
	(0.10)	(0.10)	(0.09)	(0.10)	(0.10)	(0.09)	(0.10)
Widowed	-0.290***	-0.253***	-0.258***	-0.282***	-0.305***	-0.264***	-0.304***
	(0.03)	(0.03)	(0.03)	(0.03)	(0.03)	(0.03)	(0.03)
Never Married	-0.0505	-0.0214	-0.0682	-0.0512	-0.0464	-0.0659	-0.0139
	(0.06)	(0.06)	(0.06)	(0.06)	(0.06)	(0.06)	(0.06)
Missing	-0.241*	-0.214	-0.245*	-0.273*	-0.245*	-0.212	-0.233*
	(0.11)	(0.11)	(0.11)	(0.11)	(0.11)	(0.11)	(0.11)
Medical History							
Hypertension	0.00915	0.0158	0.0108	-0.00132	-0.0373	0.0117	-0.0229
	(0.02)	(0.02)	(0.02)	(0.02)	(0.02)	(0.02)	(0.02)
Angina/Coronary Artery Disease	0.141***	0.139***	0.135***	0.141***	0.102**	0.158***	0.119***
	(0.03)	(0.03)	(0.03)	(0.03)	(0.03)	(0.03)	(0.03)
Congestive Heart Failure	0.177***	0.199***	0.216***	0.189***	0.144**	0.191***	0.121**
	(0.04)	(0.04)	(0.04)	(0.04)	(0.04)	(0.04)	(0.04)
Myocardial Infarction Question	0.00527	0.0201	0.0329	0.00670	0.00122	0.0206	-0.0132
	(0.04)	(0.04)	(0.04)	(0.04)	(0.04)	(0.04)	(0.04)
Other Heart Conditions Question	0.0508*	0.0544*	0.0578*	0.0280	0.0167	0.0543*	0.0180
	(0.03)	(0.03)	(0.03)	(0.03)	(0.03)	(0.03)	(0.03)
Stroke	0.238***	0.255***	0.282***	0.237***	0.207***	0.271***	0.165***
	(0.04)	(0.04)	(0.04)	(0.04)	(0.04)	(0.04)	(0.04)
COPD	0.197***	0.219***	0.229***	0.222***	0.162***	0.233***	0.169***
	(0.03)	(0.03)	(0.03)	(0.03)	(0.03)	(0.03)	(0.03)
Inflammatory Bowel Disease	0.258***	0.244***	0.237***	0.244***	0.230***	0.236***	0.235***
	(0.04)	(0.04)	(0.04)	(0.04)	(0.04)	(0.04)	(0.04)
Arthritis of Hip/Knee	0.0525*	0.0546*	0.0852***	-0.0154	-0.0522*	0.0592*	-0.0550*
	(0.02)	(0.02)	(0.02)	(0.02)	(0.02)	(0.02)	(0.02)
Arthritis of Hand/Wrist	0.178***	0.176***	0.172***	0.165***	0.169***	0.179***	0.159***
	(0.02)	(0.02)	(0.02)	(0.02)	(0.02)	(0.02)	(0.02)
Osteoporosis	0.0873**	0.0909**	0.105***	0.0839**	0.0783**	0.0917***	0.0750**
	(0.03)	(0.03)	(0.03)	(0.03)	(0.03)	(0.03)	(0.03)
Sciatica	0.173***	0.175***	0.178***	0.151***	0.129***	0.171***	0.136***
	(0.03)	(0.03)	(0.03)	(0.03)	(0.03)	(0.03)	(0.03)
Diabetes	0.126***	0.138***	0.145***	0.115***	0.104***	0.141***	0.0906***
	(0.02)	(0.02)	(0.02)	(0.02)	(0.02)	(0.02)	(0.02)
Any Cancer	-0.0167	-0.0169	-0.0176	-0.0149	-0.0278	-0.0128	-0.0367
	(0.03)	(0.03)	(0.03)	(0.03)	(0.03)	(0.03)	(0.03)
Hypertension_Missing	-0.131	-0.129	-0.119	-0.0964	-0.0786	-0.0997	-0.134
	(0.13)	(0.13)	(0.13)	(0.13)	(0.13)	(0.13)	(0.13)
Angina/Coronary Artery Disease_Missing	0.0740	0.0904	0.0896	0.0978	0.120	0.0961	0.109
	(0.09)	(0.09)	(0.09)	(0.10)	(0.10)	(0.09)	(0.10)
Congestive Heart Failure_Missing	-0.0295	-0.0409	-0.0567	-0.00152	-0.0670	-0.0397	-0.0420
	(0.11)	(0.11)	(0.11)	(0.11)	(0.11)	(0.11)	(0.12)
Myocardial Infarction Question_Missing	-0.0127	-0.0347	0.00136	-0.0153	-0.0110	-0.0144	-0.0472
	(0.12)	(0.11)	(0.11)	(0.12)	(0.11)	(0.11)	(0.12)
Other Heart Conditions Question_Missing	-0.111	-0.108	-0.103	-0.132	-0.130	-0.115	-0.121
	(0.11)	(0.11)	(0.11)	(0.11)	(0.11)	(0.11)	(0.12)
Stroke_Missing	0.210	0.216	0.262*	0.271*	0.258*	0.264*	0.251*
	(0.12)	(0.12)	(0.13)	(0.12)	(0.12)	(0.12)	(0.12)
COPD_Missing	0.0489	0.0521	0.00631	0.0364	0.0394	0.0397	0.0502
	(0.12)	(0.12)	(0.12)	(0.12)	(0.12)	(0.12)	(0.12)
Inflammatory Bowel Disease_Missing	0.0409	0.0125	0.00563	0.00744	-0.00791	-0.00266	0.0163
	(0.09)	(0.09)	(0.09)	(0.09)	(0.09)	(0.09)	(0.10)
Arthritis of Hip/Knee_Missing	0.224*	0.199	0.219*	0.142	0.109	0.226*	0.128
	(0.11)	(0.11)	(0.11)	(0.11)	(0.11)	(0.11)	(0.11)
Arthritis of Hand/Wrist_Missing	0.0148	0.0505	0.0181	0.0205	0.0407	0.0622	0.0193
	(0.09)	(0.09)	(0.09)	(0.09)	(0.09)	(0.09)	(0.09)
Osteoporosis_Missing	0.0810	0.0730	0.0755	0.0615	0.0386	0.0641	0.0506
	(0.10)	(0.10)	(0.10)	(0.10)	(0.10)	(0.10)	(0.11)
Sciatica_Missing	0.203*	0.206*	0.235*	0.221*	0.221*	0.213*	0.208*
	(0.10)	(0.10)	(0.10)	(0.10)	(0.10)	(0.10)	(0.10)
Diabetes_Missing	0.0563	0.0292	0.0821	0.0456	0.0476	0.0609	0.0407
	(0.13)	(0.13)	(0.13)	(0.13)	(0.13)	(0.13)	(0.13)
Any Cancer_Missing	-0.00607	-0.00263	-0.0347	-0.00965	0.0326	-0.0129	-0.00368
	(0.12)	(0.12)	(0.12)	(0.12)	(0.12)	(0.12)	(0.12)
Housing Category							
Owned or being bought by you	0	0	0	0	0	0	0
	(.)	(.)	(.)	(.)	(.)	(.)	(.)
Owned or being bought by someone in your family	0.188***	0.211***	0.230***	0.220***	0.190***	0.226***	0.159***
	(0.05)	(0.05)	(0.05)	(0.05)	(0.05)	(0.05)	(0.05)
Rented for money	0.174***	0.188***	0.201***	0.180***	0.156***	0.187***	0.141***
	(0.03)	(0.03)	(0.03)	(0.03)	(0.03)	(0.03)	(0.03)
Live without payment of rent	0.0783	0.0956	0.118	0.0861	0.0896	0.112	0.0585
	(0.08)	(0.08)	(0.08)	(0.08)	(0.08)	(0.08)	(0.08)
None of the above	0.263***	0.263***	0.271***	0.274***	0.247***	0.238***	0.234***
	(0.06)	(0.06)	(0.06)	(0.06)	(0.06)	(0.06)	(0.06)
Missing	0.203*	0.203*	0.231**	0.212*	0.202*	0.199*	0.190*
	(0.09)	(0.09)	(0.09)	(0.09)	(0.09)	(0.09)	(0.09)
BMI Category							
Underweight ( < 20)	0	0	0	0	0	0	0
	(.)	(.)	(.)	(.)	(.)	(.)	(.)
Normal (20-24)	-0.125*	-0.148**	-0.141**	-0.184***	-0.162**	-0.174***	-0.112*
	(0.05)	(0.05)	(0.05)	(0.05)	(0.05)	(0.05)	(0.05)
Overweight (25-29)	-0.196***	-0.229***	-0.220***	-0.292***	-0.272***	-0.263***	-0.202***
	(0.05)	(0.05)	(0.05)	(0.05)	(0.05)	(0.05)	(0.05)
Obese (30-34)	-0.178***	-0.210***	-0.183***	-0.311***	-0.309***	-0.242***	-0.236***
	(0.05)	(0.05)	(0.05)	(0.05)	(0.05)	(0.05)	(0.05)
Morbid Obesity (>=35)	-0.219***	-0.248***	-0.187**	-0.364***	-0.399***	-0.265***	-0.321***
	(0.06)	(0.06)	(0.06)	(0.06)	(0.06)	(0.06)	(0.06)
Missing	-0.0374	-0.0587	-0.0290	-0.128	-0.124	-0.103	-0.0830
	(0.09)	(0.09)	(0.09)	(0.09)	(0.09)	(0.09)	(0.09)
Current Smoker	0.289***	0.304***	0.287***	0.316***	0.260***	0.309***	0.275***
	(0.03)	(0.03)	(0.03)	(0.03)	(0.03)	(0.03)	(0.03)
Current Smoker_Missing	-0.0913	-0.0803	-0.0980	-0.0649	-0.0640	-0.0752	-0.0653
	(0.13)	(0.13)	(0.13)	(0.13)	(0.13)	(0.13)	(0.13)
Bone Density Test for Osteoporosis	-0.0854**	-0.0979***	-0.0981***	-0.0960***	-0.0904**	-0.107***	-0.0699*
	(0.03)	(0.03)	(0.03)	(0.03)	(0.03)	(0.03)	(0.03)
Bone Density Test for Osteoporosis_Missing	0.188*	0.163	0.196*	0.186*	0.199*	0.193*	0.178*
	(0.09)	(0.09)	(0.09)	(0.09)	(0.09)	(0.09)	(0.09)
Urine Leakage	0.187***	0.186***	0.197***	0.150***	0.148***	0.178***	0.133***
	(0.02)	(0.02)	(0.02)	(0.02)	(0.02)	(0.02)	(0.02)
Urine Leakage_Missing	0.361***	0.349***	0.359***	0.334***	0.330***	0.342***	0.317***
	(0.09)	(0.09)	(0.09)	(0.09)	(0.09)	(0.09)	(0.09)
Talked with Doctor About Physical Activities	-0.134***	-0.143***	-0.156***	-0.143***	-0.134***	-0.145***	-0.122***
	(0.02)	(0.02)	(0.02)	(0.02)	(0.02)	(0.02)	(0.02)
Talked with Doctor About Physical Activities_Missing	0.0674	0.0697	0.0695	0.0615	0.0617	0.0799	0.0702
	(0.07)	(0.07)	(0.07)	(0.07)	(0.07)	(0.07)	(0.07)
ADL Items							
Bathing	2.995***						
	(0.03)						
Dressing		3.193***					
		(0.04)					
Eating			3.538***				
			(0.05)				
Getting In/Out of Chairs				2.256***			
				(0.03)			
Walking					2.072***		
					(0.02)		
Using the Toilet						3.274***	
						(0.04)	
ADL Scale							
0							0
							(.)
1							1.391***
							(0.04)
2							2.558***
							(0.06)
3							4.352***
							(0.12)
4							6.358***
							(0.17)
5							8.541***
							(0.24)
6							9.799***
							(0.22)
Benefit Duration Less than 1 Year	-0.0510*	-0.0541*	-0.0634*	-0.0447	-0.0378	-0.0559*	-0.0368
	(0.03)	(0.03)	(0.03)	(0.03)	(0.03)	(0.03)	(0.03)
Plan Duration Less than 5 Year	-0.0145	-0.0199	-0.0194	-0.0198	-0.0153	-0.0183	-0.0185
	(0.03)	(0.03)	(0.03)	(0.03)	(0.03)	(0.03)	(0.03)
Year Trend	-0.0581***	-0.0583***	-0.0600***	-0.0561***	-0.0575***	-0.0572***	-0.0556***
	(0.01)	(0.01)	(0.01)	(0.01)	(0.01)	(0.01)	(0.01)

Notes: This is the full results for Panel A of Table 3 with adjustment for covariates listed in Table 1 except baseline PCS, MCS and PUD. **p*<0.1, ***p*<0.05, ****p*<0.01

## Appendix 5

**Table 8:** The effect of the onset of ADL difficulties on SF-12V Items

	Items for Constructing PCS						Items for Constructing MCS					
	Physical Functioning		Role Physical		Body Pain	Gen Health	Vitality	Social	Role Emotional		Mental Health	
	SF-2	SF-3	SF-4	SF-5	SF-8	SF-1	SF-10	SF-12	SF-6	SF-7	SF-9	SF-11
Bathing	0.218***	0.161***	0.232***	0.244***	0.200***	0.147***	0.185***	0.258***	0.196***	0.192***	0.153***	0.185***
	(0.004)	(0.004)	(0.007)	(0.007)	(0.006)	(0.005)	(0.007)	(0.006)	(0.006)	(0.006)	(0.007)	(0.007)
Dressing	0.193***	0.142***	0.213***	0.225***	0.193***	0.139***	0.170***	0.238***	0.190***	0.186***	0.145***	0.170***
	(0.005)	(0.005)	(0.008)	(0.008)	(0.007)	(0.005)	(0.009)	(0.007)	(0.006)	(0.006)	(0.008)	(0.009)
Eating	0.111***	0.082***	0.135***	0.140***	0.115***	0.096***	0.115***	0.165***	0.146***	0.143***	0.109***	0.115***
	(0.007)	(0.007)	(0.011)	(0.011)	(0.010)	(0.008)	(0.012)	(0.010)	(0.009)	(0.009)	(0.012)	(0.012)
Getting In/Out of Chairs	0.231***	0.200***	0.248***	0.261***	0.251***	0.152***	0.203***	0.244***	0.195***	0.200***	0.152***	0.203***
	(0.003)	(0.003)	(0.005)	(0.005)	(0.004)	(0.004)	(0.005)	(0.004)	(0.004)	(0.004)	(0.005)	(0.005)
Walking	0.307***	0.271***	0.315***	0.336***	0.319***	0.188***	0.251***	0.287***	0.216***	0.223***	0.170***	0.251***
	(0.002)	(0.003)	(0.004)	(0.004)	(0.004)	(0.003)	(0.004)	(0.003)	(0.003)	(0.003)	(0.004)	(0.004)
Using the Toilet	0.154***	0.121***	0.177***	0.185***	0.160***	0.114***	0.139***	0.204***	0.165***	0.167***	0.124***	0.139***
	(0.005)	(0.006)	(0.009)	(0.009)	(0.008)	(0.006)	(0.010)	(0.008)	(0.007)	(0.007)	(0.009)	(0.010)

Notes: All SF-12 items are recoded such that higher scores represent worse HRQoL. Each cell is a separate point estimation of one ADL item on one SF-12V item. The coefficients are standardized coefficients (i.e. the beta coefficients). We utilized the same model specification as our main analysis. Standard errors are in the parentheses. **p*<0.1, ***p*<0.05, ****p*<0.01

## Appendix 6

**Table 9:** The factor analysis of SF-12V items and CDC unhealthy days items

	Physical Health Factor	Mental Health Factor
Panel A (N=407285, KMO=0.92)		
SF-1: General Health Question	0.639	
SF-2: Moderate Activities Question	0.860	
SF-3: Climbing Several Flights of Stairs Question	0.861	
SF-4: Physical Health Limiting Amount Accomplished Question	0.813	
SF-5: Physical Health Limiting the Kind of Activities Question	0.851	
SF-6: Emotional Problems Limiting Amount Accomplished Question		0.767
SF-7: Emotional Problems Limiting Carefulness Question		0.685
SF-8: Pain Interfering with Work Question	0.706	
SF-9: Calm and Peaceful Question		0.660
SF-10: Lots of Energy Question	0.590	
SF-11: Downhearted and Blue Question		0.769
SF-12: Amount of Time Health Interfering with Social Activities		0.555
CDC Physically Unhealthy Days	0.450	
CDC Mentally Unhealthy Days		0.829
Panel B (455230, KMO=0.64)		
PCS	-0.714	
MCS		0.857
CDC Physically Unhealthy Days	0.960	
CDC Mentally Unhealthy Days		-0.917

Notes: All SF-12 items are recoded such that higher scores represent worse HRQoL

## Abbreviations

ADL: Activities of Daily Living
CDC: Center for Disease Control and Prevention
HRQoL: Health-related Quality of Life
MAOs: Medicare Advantage Organizations
MCS: Mental Health Component Scores
M-HOS: Medicare Health Outcomes Survey
MUD: Mentally Unhealthy Days
OLS: Ordinary Least Square
PCS: Physical Health Component Scores
PUD: Physically Unhealthy Days
SF-12V: Short-Form 12-Item Questionnaire for Veterans
ZINB: Zero-Inflated Negative Binominal Regression
